# Catchability Pattern of Cartilaginous Fishes with an Updated Their Diversity and Distribution Along the Egypt’s Mediterranean Coast: Conservation and Bycatch Challenges

**DOI:** 10.3390/ani16111730

**Published:** 2026-06-04

**Authors:** Mahmoud M. S. Farrag, Mohamed Adel, Mennatallah M. A. El-Geddawy, Fabrizio Serena

**Affiliations:** 1 Zoology Department, Faculty of Science, Al-Azhar University (Assiut), Assiut 71524, Egypt; m_mahrousfarrag@yahoo.com; 2 Independent Researcher, Alexandria 21648, Egypt; mohammad.thabit@gmail.com; 3 Food Science & Technology Department, Faculty of Agriculture, Assiut University, Assiut 71526, Egypt; menatalah.elanwar@agr.aun.edu.eg; 4 Institute for Marine Biological Resources and Biotechnology, National Research Council (CNR-IRBIM), Via Vaccara, 61, 91026 Mazara del Vallo, Italy

**Keywords:** sharks and rays, diversity, distribution, new records, catchability, species composition, Mediterranean Sea, Egypt, Bycatch and conservation

## Abstract

The diversity and fisheries status of cartilaginous species along the Egyptian Mediterranean coast were investigated up to 2025. A total of 69 species were recorded, comprising 2 chimaeras, 41 sharks, and 26 rays and skates, including new records such as *Cetorhinus maximus*, *Odontaspis ferox*, and *Dalatias licha*. The coastline was divided into five zones (A–E), with the highest species diversity observed in Zone D (western Alexandria to Matrouh), followed by Zone C (off Alexandrian coast). The total landed catches and bycatch from various fishing gear were estimated from 2023 to 2025. In 2025, total annual catch reached 665,820 kg. Damietta recorded the highest landings in 2025 (274,350 kg; 41.20%), whereas Port Said had the lowest. Among elasmobranchs, *Glaucostegus cemiculus* accounted for the highest catch (109,100 kg; 16.39%), followed by *Carcharhinus plumbeus* (95,500 kg; 14.34%). Spring season showed the highest catchability percentage over the three-year period. Alexandria serves as the prime market for elasmobranchs, receiving additional quantities from the Gulf of Suez and the Red Sea, along with Damietta. Port Said occasionally shares this role. The findings highlight the need for conservation, particularly in Zone D, as it is a hotspot for diversity, nursery, and reproductive activity. Strengthened control of fishing and trade is recommended from April to October, with emphasis on the period of April–May. Additionally, raising awareness among fishers is essential to reduce the capture of juveniles and pregnant individuals, especially during the periods of March–May and October–December.

## Simple Summary

Filling in the gap in cartilaginous fish’s status along the Egyptian Mediterranean coast was conducted historically up to 2025. The diversity consisted of 69 species (2 chimaeras, 41 sharks, and 26 rays and skates), including new species records (*Cetorhinus maximus*; *Odontaspis ferox*; and *Dalatias licha*). As the coast was divided into five zones (A–E), the species was the highest in Zone D (western Alexandria to Matrouh), followed by Zone C (off the Alexandrian coast). From the landing sites, the total catch was estimated during 2023–2025. Specifically, for 2025, it amounted to 665,820 kg. The landing site with the highest amount was Damietta (274,350 kg: 41.20%) during 2025, while Port Said had the lowest one. Among the recorded elasmobranchs, *Glaucostegus cemiculus* recorded the highest catch during 2025 (109,100 kg, 16.39%), followed by *Carcharhinus plumbeus* (95,500 kg, 14.34%). Alexandria is the primary market of elasmobranchs. This study reflected the required conservation, mostly with Zone D (western Alexandria) as a hot spot area for nursery and reproduction. Control on fishing and trading is recommended from April to October.

## 1. Introduction

Globally, Chondrichthyes are a source of interest for human concerns and play a vital role in the biological balance and environmental support. They constitute three categories: sharks, batoids (rays and skates), and chimaeras. Worldwide, their number reached 1282 species, divided into 537 shark species in 34 families, 689 batoid species in 20 families, and 56 species of Holocephali belonging to 3 families [1,2]. The Mediterranean Sea stands as a biodiversity hotspot, hosting approximately 17,000 marine species [3], of which more than 600 (3.3%) are non-native. The taxonomic status of Chondrichthyes in the Mediterranean Basin sometimes faces additional challenges due to fishing pressure and the introduction of non-indigenous species through various pathways [4,5], which are sometimes morphologically similar to native species. At least 53% of the Chondrichthyes species found in the Mediterranean are classified by the IUCN (International Union for Conservation of Nature) as ‘vulnerable’ and ‘endangered [6,7], and a large proportion of species (20%) are still classified as ‘Data-Deficient’.

In the Mediterranean and Black seas, the Food and Agriculture Organization (FAO) has reported 48 shark species across 20 families and 6 orders (Hexanchiformes, Squaliformes, Squatiniformes, Lamniformes, Echinorhiniformes, and Carcharhiniforms) [8]. This guide contains an illustrated key to the orders, families, genera, and species of the Chondrichthyes in the Mediterranean and Black seas (FAO fishing area 37), currently represented by 38 sharks, 48 batoids, and 2 chimaeras. Alarmingly, 24% of the shark, skate, and ray species are considered threatened with extinction by the IUCN Red List’s Shark Specialist Group, highlighting the urgent need for conservation efforts [6]. The increase in the consumption of shark products, along with the shark’s fishing vulnerabilities, has led to a decrease in certain shark populations [9].

The Egyptian Mediterranean waters host 13 species from the Carcharhiniforms order, distributed among 4 families: Scyliorhinidae, Triakidae, Carcharhinidae, and Sphyrnidae [10]. Recently, the number of families has increased to five, by reclassifiying *Galeus melastomus* under the family Pentanchidae. Earlier efforts in this sector date back to the studies [11,12] that investigated the biological aspects of the Triakidae family and the shark species composition along the coast of Alexandria. Allam [13] revised the order Hypotremata along the Mediterranean coast off Alexandria with special reference to the family Dasyatidae. Limited studies were conducted from the early 2000s onwards, with significant gaps addressed by several authors [14,15,16,17,18]. However, these studies focused on specific species in certain areas along the coast. Notably, deep-sea fisheries have gained attention in the Mediterranean, and studies by Ibrahim et al. [19] and Farrag [20,21] have contributed to our knowledge about elasmobranchs beyond 400 m in depth. However, the data regarding elasmobranchs still remain insufficient due to some factors such as the high costs of shark bait, Egyptian laws prohibiting shark fishing activity (leading to a low number of fishers targeting elasmobranchs), a low number of deepwater trawlers catching deeper species, and the topography of the shallow delta region along the coast. Collectively, these factors negatively affect the data obtained on elasmobranchs. In recent years, elasmobranchs, particularly sharks, have become an important subject along the Mediterranean Sea, especially in relation to the change in diversity driven by climate change and migration. Therefore, this study aimed to provide an update on the elasmobranchs’ diversity, new records, distribution, and fisheries status along the entire Egyptian coast of the Mediterranean Sea in order to fill this knowledge gap, while highlighting the bycatch challenge of sharks and rays in the region, and suggesting future conservation measures and bycatch challenges for this important group of animals.

## 2. Materials and Methods

### 2.1. Study Area and Sampling

**Figure 1:**
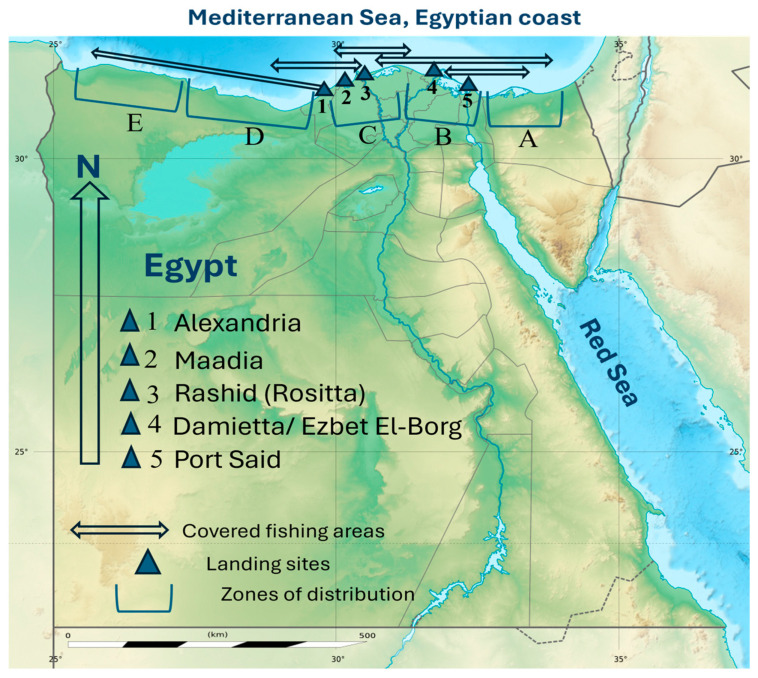
Map of the major landing sites receiving the elasmobranch catch along the Egyptian Mediterranean coast (1. Alexandria/Anfoushy; 2. Maadia; 3. Rashid; 4. Damietta/Ezbet El-Borg; and 5. Port Said). Arrows indicate the fishing range of fishers at each site.

This study was conducted to assess elasmobranchs along the Egyptian Mediterranean coast which extends for a length of about 1200 km, spanning from surface waters to depths of 800 m (Figure 1). Data were collected from various sources, including landing records and bycatch, direct diving, onboard observations, and published literature, to depict a comprehensive picture of elasmobranchs in the catch up to 2025. The data obtained represented various types of fishing gear including drifting and bottom longlines, bottom trawlers, purse seiners, gills, and trammel nets, as well as some minor fishing operations.

### 2.2. Data and Sample Processing

Data of elasmobranchs were gathered from all sources mentioned above, supplemented by direct onboard surveys. In addition, weekly visits were conducted the major landing sites (Alexandria/Anfoushy–Maadia–Rashid–Damietta/Ezbet El-Borg–Port Said). These sites were chosen as they receive the majority of landed elasmobranchs caught by various fishing boats operating along the entire coastline and effectively covering the whole area (Figure 1).

Several smaller harbors are also distributed along the coast, including Matrouh and El-Max in the western part of Alexandria; Baltim harbor located between Rashid and Ezbet El-Borg in the central region; and Arish Harbor in the eastern sector near the Gaza border. These harbors have various registered various efforts of fisheries activities. However, they considered a minor importance for landings elasmobranchs due to their fishing operating, lower variety of vessels and marketing of elasmobranchs. Otherwise, some harbors such as Baltim landed its catch in Rashid and Maddia. Therefore, the major sites will be suitable filling the gap of elasmobranchs knowledge based on covering fishing areas and landed catch accompanied by marketing activity.

The collected samples onboard fishing vessels and landings were photographed by Digital camera and smart phone to be utilized for identification and later description of fisheries status. The specimens were identified following the keys and validations provided by Farrag [20,21], and Barone et al. [8], in addition to the Online Database of Eschmeyer’s Catalog of Fishes, together with FishBase [22], and World Register of Marine Species (WORMS) [23].

The species diversity was reported historically from other works of literature and present study from 2020 up to today to confirm the presence of species in the same area as recorded before or to extend it to other areas in terms of distribution, and to record new occurrences as an update in diversity. For species distribution, the study divided the Egyptian coast into five zones from A to E (A: area from Al-Arish to Port Said at the border at the eastern border of Egypt; B: east of the Delta, extending slightly from Port Said to beyond Rashid; C: located off the Delta region to Alexandria—typically a region of shallow waters ranging from 150 m to 200 m depth; D: from Alexandria to Marsa Matrouh westward; and E: located at the western part of Egypt to the Sallum region at the border with Libya (Figure 1).

While the concern for catchability and relative fisheries was were estimated during 2023, 2024, and 2025, with regard to the concern for catchability and the relative fisheries, weekly visits were made to the five major landing sites, and questionnaires were conducted with fish traders to ensure the collection of comprehensive fisheries data from late 2020 up to today, with an emphasis on the time during 2023–2025 for catch data. Fishing gear, dates, number of samples, catch weigh per species, and sizes as available were reported. There were no regular fishing operations that targeted elasmobranchs with certain or general vessels due to the prohibition of their fishing by the Egyptian laws. Therefore, the catch of elasmobranchs was estimated from the major landing sites to ensure the reporting of all elasmobranchs caught by various types of fishing gear. In addition to that, total weight and data of catch per species were obtained from the direct observation of weights, logbooks of the traders in landing sites, and from vessel owners. Besides the visits to sites, a follow-up of the landed catch in various sites was applied every two days during 2023–2025 via questionnaire with colleagues and fishers. Data on the type of catch (target, bycatch, or discard) were meticulously documented to understand the status of the fish and to serve as a guide to future management. Catch and number of days were reported in general for all landed elasmobranchs during 2023–2025 relative to total landed catch; then, the percentage of each fishing gear was detected.

### 2.3. Fishing Gears and Fisheries Bycatch

Most landed elasmobranchs were caught as bycatch from various types of fishing gear such as deeper trawlers as described [19,20], and shallow to moderate trawlers [17]. Other fishing gear included purse seines and longlines, which were mentioned and described by Akel [24] and Farrag [25]. There are minor types of fishing gear such as a few artisanal small boats and trolling and recreational activity landed very small quantities with a low variety of species. Generally, the types of fishing gear specifically targeting elasmobranchs are few and only occasionally working, these are longlines and gillnets with modifications in their features as follows.

#### 2.3.1. Longlines (Bottom and Drifting)

**Figure 2:**
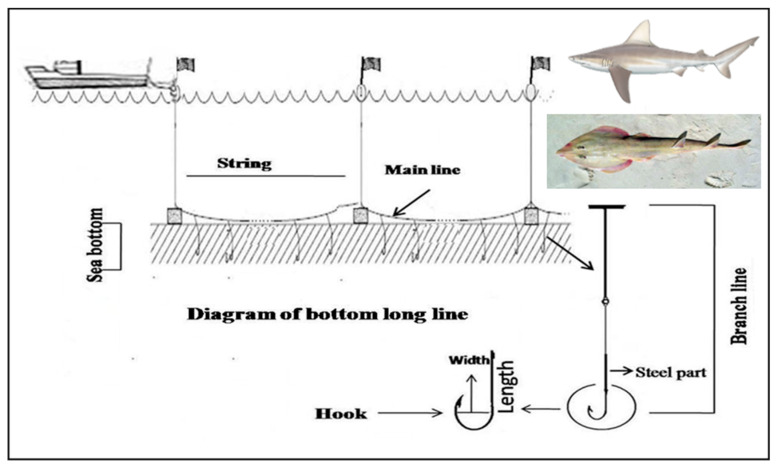
Diagram of a modified longline that catches sharks and rays in the Egyptian Mediterranean coast (occasional times/not regular).

This gear catches the elasmobranchs usually as bycatch—it can be selective to these species after some modifications as follows: it consists of number of strings that are joined together to be longer (up to 1000 m), each one consisting of a horizontal mainline extending in length from 150 to 300 m as a monofilament (2.5 mm diameter/200–250 TD). Each string contains around 75 to 150 vertical branched lines (1.5 to 2.5 m in length/height with 1.75 mm diameter) (150–175 TD) and the distance between every two successive branches is 2–3 m; then, it ends with baited hooks. Hooks vary in number between 500–700 and are J-shaped (40 to 80 mm in length, and 20 to 40 mm in width). The hooks with code numbers are 08; 010; and 013 are used for large sharks such as sandbar, big nose, silky, and bluntnose six gill shark, among others. Hooks with code numbers 7 and 8 mostly used for small sharks such as cat and dog sharks that have smooth teeth. Moreover, there is a specific steel part (30–40 cm) attached to the branch lines before hooks that is used for large sharks and rays to avoid the cutting of the branched line by their sharp teeth (Figure 2).

Bait is mostly in the form of small tuna’s slices and cephalopods (*Octopus* spp. and *Sepia* spp.). The longline is set on the seabed; its fishing operations take place mostly near sandy areas with water depths from 6 to 150 m. For small sharks and guitar sharks, the same above-mentioned gear is used, while the thickness of the mainline will be decreased to 1.2 mm diameter/120 TD and the branch line to 0.75 mm diameter/75 TD. The bait is sardine species.

#### 2.3.2. Gillnet

**Figure 3:**
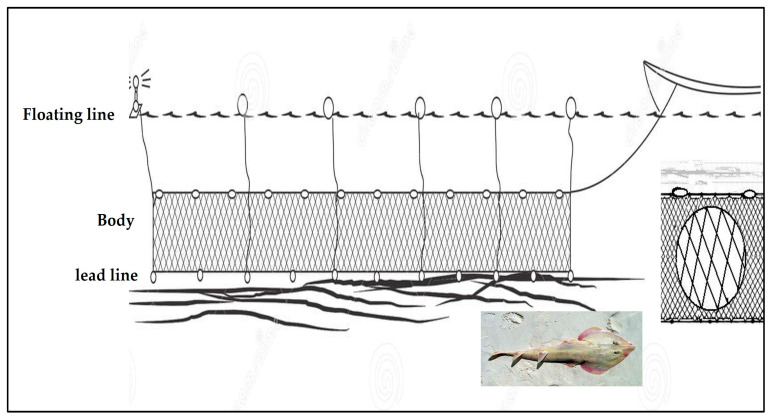
Diagrammatic shape representing a typical gillnet used along the Egyptian Mediterranean waters with some modifications for sharks and rays.

The gillnet is the second gear catching sharks and rays, both as target species and bycatch. It is one-layer monofilament or multifilament with mesh size ranging from 80 to 120 mm with silk filament (36 TD) for sharks, while, for guitar shark, the mesh size ranged from 50 to 100 mm with silk filament (24 TD). The net body is around 8 m in height for sharks and 3.5 m for guitarfish. The net length ranged from 400 to 950 m (average of 600 m), depending on the type of boat, engine power, number of fishers, and season. The floating line is made of plastic (6 mm). It is operated in sandy and rocky grounds with a depth ranging from 10 to 60 m (Figure 3).

The length range of and variations in elasmobranchs were recorded whenever possible to be used as fisheries’ keys and measures. The length was recorded for permitted and available specimens in landings sites as well as in the trading places. There were struggles and difficulties while measuring all individual elasmobranchs due to some restrictions and the high weight of some species. Statistical analysis was applied using Microsoft Excel to detect the average and other related items. Microsoft PowerPoint was used to adjust the figures and maps.

## 3. Results

### 3.1. Cartilaginous/Elasmobranchs Diversity

**Figure 4:**
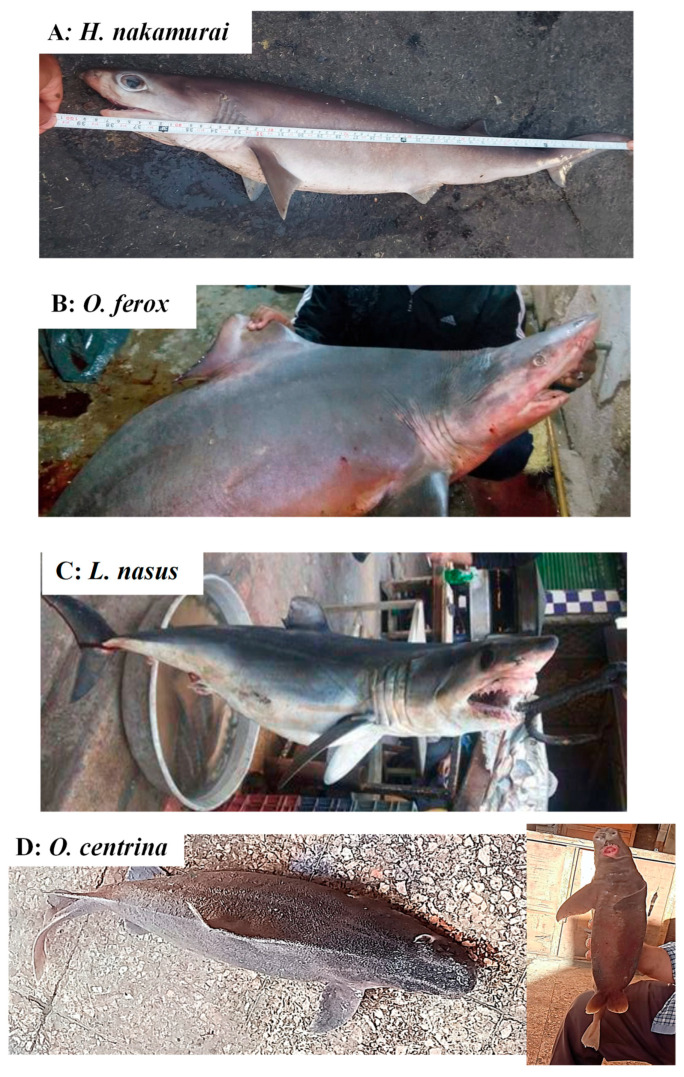

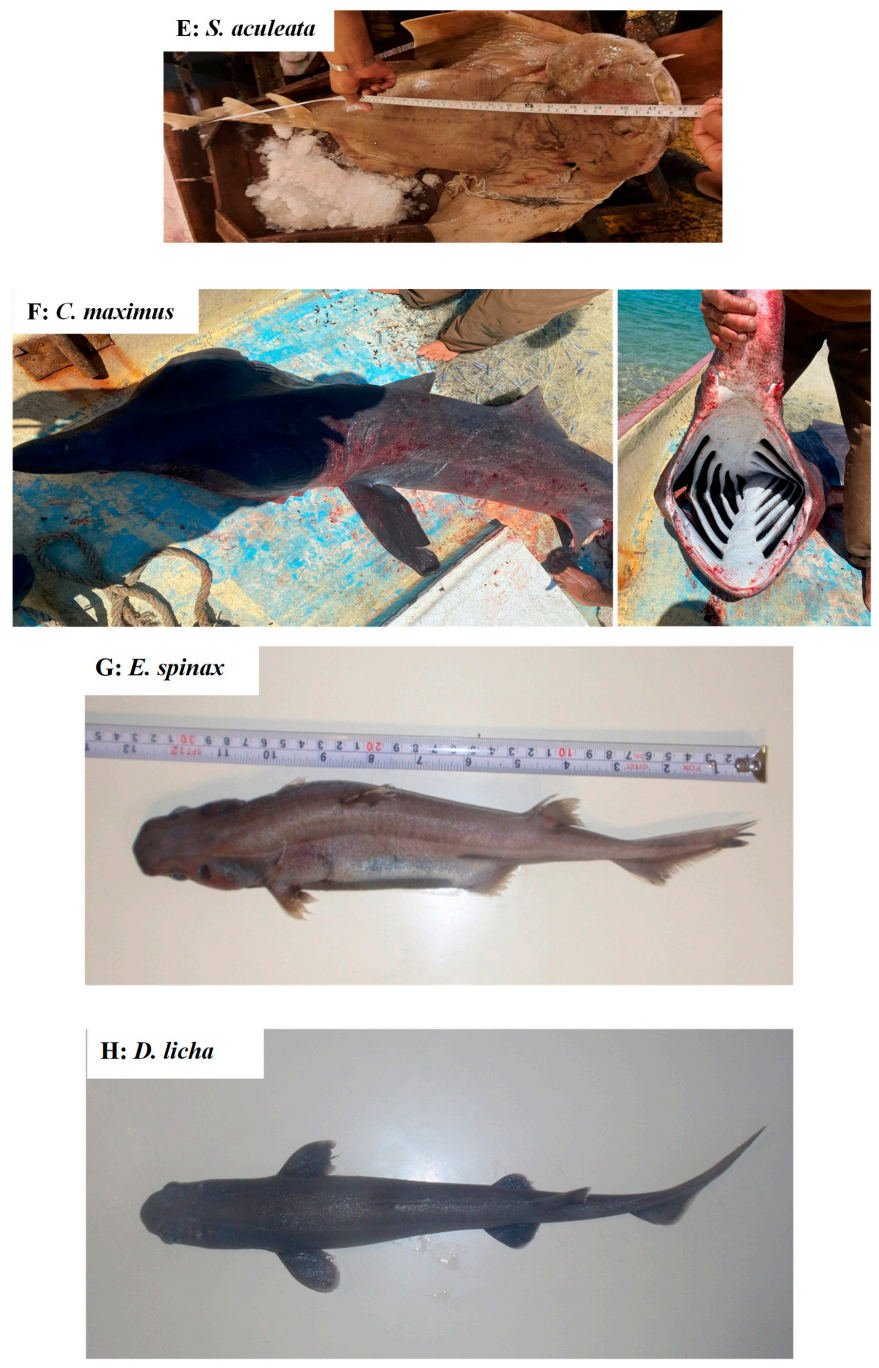
Some rare species of elasmobranchs reported in the current study for confirmation of their presence, with photos of new records (*Cetorhinus maximus*; *Odontaspis ferox*; and *Dalatias licha*), along the Egyptian Mediterranean coast: (**A**) *H. nakamurai*; (**B**): *O. ferox* taken by Mohammed Nabil; (**C**) *L. nasus*; (**D**) *O. centrina*; *(***E**) *S. aculeata*; (**F**) *C. maximus* taken by Osama El-Mandrawy; (**G**): *E. spina.*; and (**H**) *D. lic*.

This study has documented the updated list of chondrichthyans along the Egyptian Mediterranean coast, encompassing both the historical and current species records. Table 1 presents a breakdown of the cartilaginous fish species, totaling 69 species across three categories: Chimaeridae (2 species/1 family), sharks (41 species/18 families), and rays and skates (26 species/9 families). These species are distributed totally across 28 families, with Carcharhinidae (8 species) and Rajiidae (8 species) being the most abundant families. Notably, each of the nine families (Alopiidae, Cetorhinidae Dalatiidae, Echinorhinidae, Etmopteridae, Odontaspididae, Oxynotidae, Pentanchidae, and Somniosidae) included a single shark species, while one family (Gymnuridae) represented a single species of rays and skates. Family carcharhinidae is a common category of sharks and represented by species of *Carcharhinus altimus* (Springer, 1950), *Carcharhinus brachyurus* (Günther, 1870), *Carcharhinus brevipinna* (Valenciennes, 1839), *Carcharhinus falciformis* (Bibron, 1839), *Carcharhinus limbatus* (Valenciennes 1839), *Carcharhinus obscurus* (Lesueur, 1818), *Carcharhinus plumbeus* (Nardo, 1827) and *Prionace glauca* (Linnaeus, 1758). On the other hand, Rajiidae considered also a common category of chondrichthyans and is listed by *Dipturus oxyrinchus* (Linnaeus, 1758), *Leucoraja circularis* (Couch, 1838), *Raja asterias* (Delaroche, 1809), *Rostroraja alba* (Lacepède, 1803), *Raja clavata* (Linnaeus, 1758), *Raja miraletus* (Linnaeus 1758), *Raja montagui* (Fowler, 1910), *and Raja radula* (Delarochae, 1809). In this Table 1, the great white shark was reported by Akel and Karachle [10], with no further confirmation until now onboard or in landing sites. Figure 4 shows various species including newly reported here, such as *Cetorhinus maximus* (Gunnerus, 1765); *Odontaspis ferox* (Risso, 1810); and *Dalatias licha* (Bonnaterre, 1788). Other considered rare species such as *Hexanchus vitulus* Springer & Waller 1969, *Lamna nasus* (Bonnaterre, 1788), and *Oxynotus centrina* (Linnaeus, 1758) were reported as confirmation for those reported previously wether in the same area or expanded in other areas.

### 3.2. Mapping and Distribution

**Figure 5:**
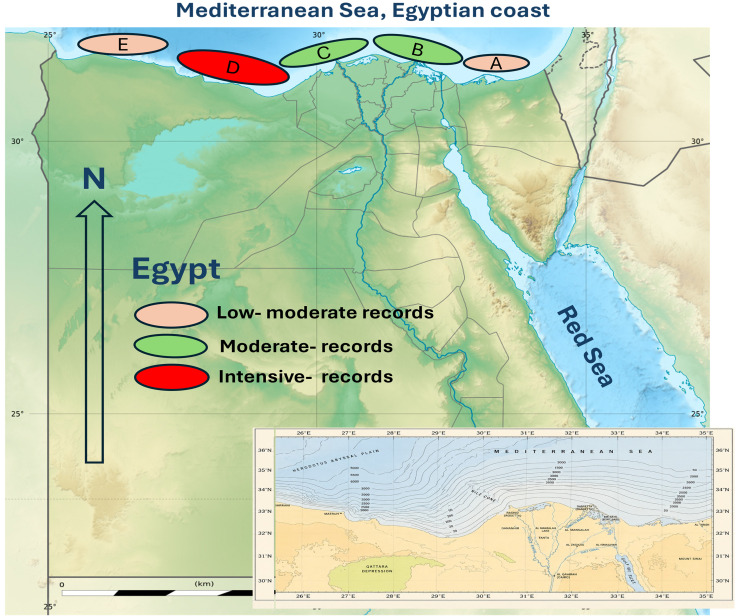
Zones A–E showing the cartilaginous fish distribution/intensity of reports along the Egyptian Mediterranean coast (green circles: moderate records; red circle: intensive records; and pink circles: low–moderate records with low fishing activity). Attached contour map showing depths ranges along the coast.

Elasmobranchs are distributed along the entire Egyptian Mediterranean coast, from Al-Arish to Sallum, across a range of depths within five defined zones (A–E; Figure 5), exhibiting distinct spatial patterns. As shown in Table 1 and Figure 5, Zones B and C support the highest species richness, coinciding with intense fishing activity using multiple gear types. Among these, Zone C (west of Alexandria) ranks highest in overall abundance. In contrast, Zones A and E, located at the eastern and western borders of Egypt, respectively, show the lowest recorded species numbers, likely reflecting limited fishing effort in these areas. From the survey, the Delta regions (Zones B and C) are characterized by shallow, muddy substrates that provide suitable habitats for a wide range of species, particularly rays and skates, resulting in the highest observed diversity. Zone D, by comparison, exhibits the greatest intensity of elasmobranch occurrence, especially for guitar fish and large shark species. This pattern is likely related to its proximity to Zone C; it is also supported by a relative near deeper bottom environment, which supports species associated with deeper habitats. Consequently, zone C may rank second in overall elasmobranch diversity. Clear spatial variation in species composition was observed among zones. Some species, including the shortfin mako (*I. oxyrinchus*), sandbar shark (*C. plumbeus*), silky shark (*C. falciformis*), and blackchin guitarfish (*Glaucostegus cemiculus* (Geoffroy St. Hilaire, 1817)), were widely distributed across multiple zones and represent key components of fisheries bycatch. In addition, several species were recorded in new zones, extending their previously known distributions.

Otherwise, the small dog sharks and catsharks, together with large deeper sharks such as *Hexanchus griseus* (Bonnaterre, 1788), were abundant in Zones B and D, where suitable and deeper habitats occur. Table 1 also highlights species of conservation concern, including the critically endangered angelshark (*Squatina squatina*) and less abundant species such as the smalltooth sand tiger shark (*O. ferox*) and the angular roughshark (*O. centrina*), all of which are now considered rare in the study area. Zone C, with its muddy and shallow habitats, influenced the distribution and occurrences of elasmobranchs, particularly large pelagic sharks. However, this area included small rays and small-sized pelagic species such as the shortfin mako.

### 3.3. Fisheries Situation

#### 3.3.1. Catchability and Species Composition

**Figure 6:**
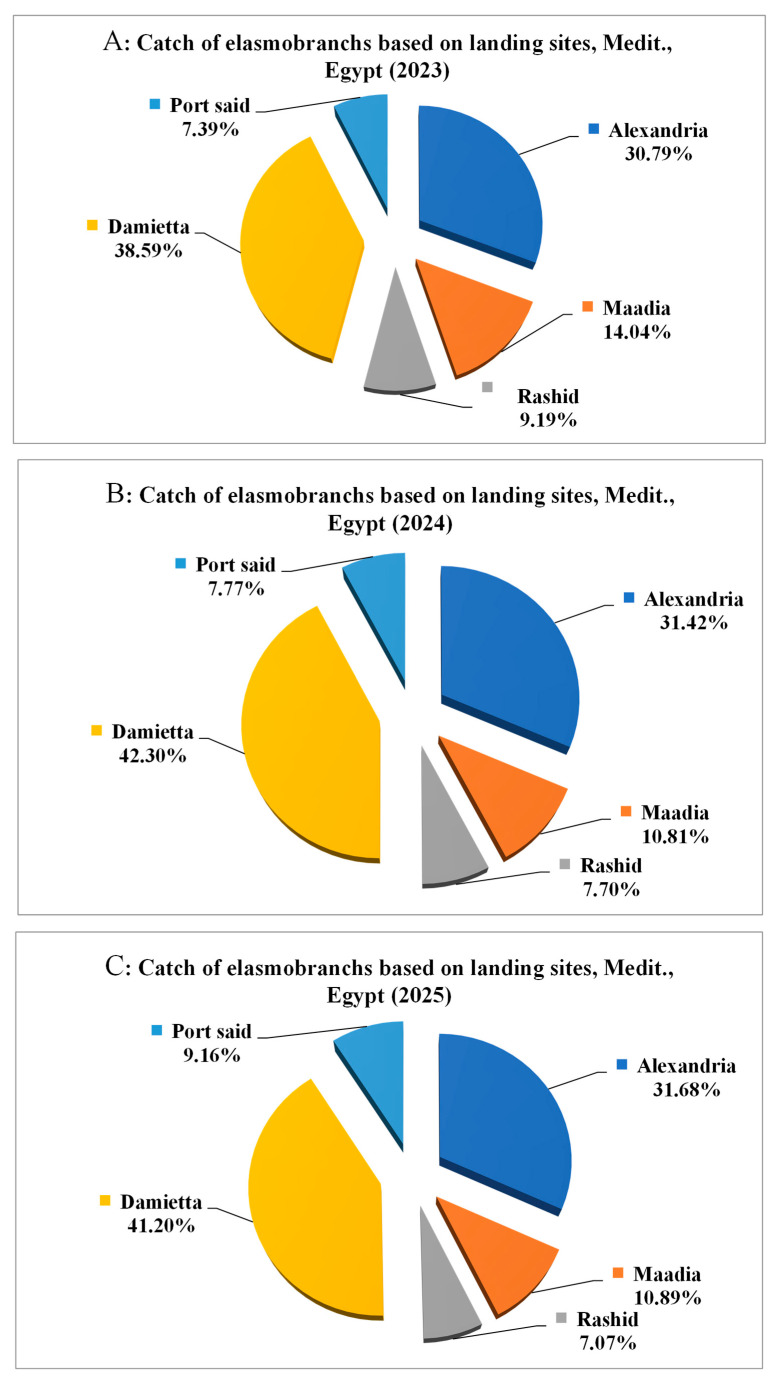
The estimated catch of elasmobranchs according to major landing sites along the Egyptian Mediterranean coast during 2023–2025: (**A**) 2023, (**B**) 2024, and (**C**) 2025.

The catchability and exploitation of elasmobranchs were estimated from five major landing sites which receive the catches from various types of fishing gear. The total estimated catch was 541,450 kg, 611,645 kg, and 665,820 kg for elasmobranchs along the Egyptian Mediterranean coast during 2023, 2024, and 2025, respectively (Table 2 and Figure 6).

From the same table, the annual variations in species composition illustrated, the highest annual catches were estimated for the blackchin guitarfish *G. cemiculus*: 92,000 kg (16.99%), 89,500 kg (14.63%), and 109,100 kg (16.39%) from 2023 to 2025, respectively. These values were followed by the catch of *C. plumbeus*, which amounted to 76,000 kg (14.04%), 88,000 kg (14.39%), and 95,500 kg (14.34%) during 2023, 2024, and 2025, respectively. The same table illustrated that the species *C. altimus*, *C. falciformis*, *Rhinobatos rhinobatos* (Linnaeus, 1758), and *H. griseus* were reported to have high levels of the estimated catch during the years 2023–2025.

For pure pelagic sharks, such as the blue shark *P. glauca*, a catch of 7800 kg with percentage of 1.44% was recorded during 2023, which then increased to 9000 kg with a percentage of 1.48% during 2024 and 11,100 kg (1.67%) during 2023 to 2025, respectively. The catch of shortfin mako *Isurus oxyrinchus* (Rafinesque, 1810) was 92,000 kg with a percentage of 1.7% during 2023; then, it increased to 10,900 kg (1.78%) during 2024 and 11,700 kg (1.76%) during 2025. The large pelagic ray *Mobula mobular* (Bonnaterre, 1788) catch was 36,000 kg with a percentage of 6.65% during 2023, and 34,350 kg (5.58%) during 2024, and 32,900 kg (4.94%) during 2025, while the other species showed a decrease in their percentages.

**Figure 7:**
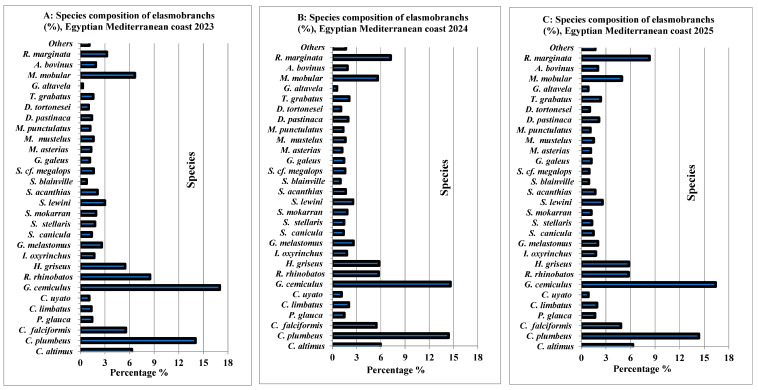
Annual species composition of the major landed elasmobranchs (%) along the Egyptian Mediterranean coast estimated from the major landing sites during 2023–2025 with bycatch fisheries and few targeting boats: (**A**) 2023, (**B**) 2024, and (**C**) 2025.

The regional variations of the annual species catchability are presented in Table 2 and Figure 7. The highest landing site during 2023 was Damietta (208,920 kg; 38.59%), followed by 166,750 kg (30.79%) for Alexandria, while the lowest catch was estimated at Port Said (40,000 kg; 7.39%). The same trend has been estimated during 2024 and 2025, where the highest landing site was Damietta with 258,725 kg (42.30%) and 274,350 kg (41.20%), followed by 192,195 kg (31.42%) and 210,900 kg (31.68%) for Alexandria during 2024 and 2025, respectively, while the lowest catch was estimated at Port Said at 47,530 kg (7.77%) and 61,020 (9.16%) for the same period, respectively.

Seasonal variation in the general catchability pattern during 2023–2025 illustrated that spring ranked first in elasmobranch catches, particularly during April and May, accounting for 35%, 42%, and 38% of the total estimated catch during 2023, 2024, and 2025, respectively. Summer ranked second, contributing 28%, 33%, and 32% during the same years. In contrast, winter consistently recorded the lowest catchability, representing 17% in 2023, 10% in 2024, and 11% in 2025. During autumn, elasmobranch catches constituted 20%, 15%, and 19% of the total estimated catch in 2023, 2024, and 2025, respectively.

**Figure 8:**
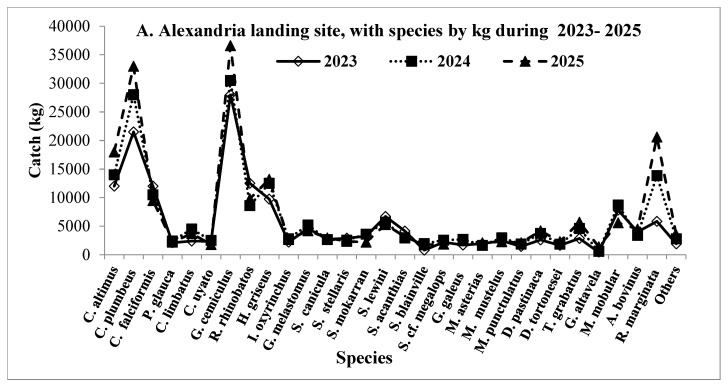

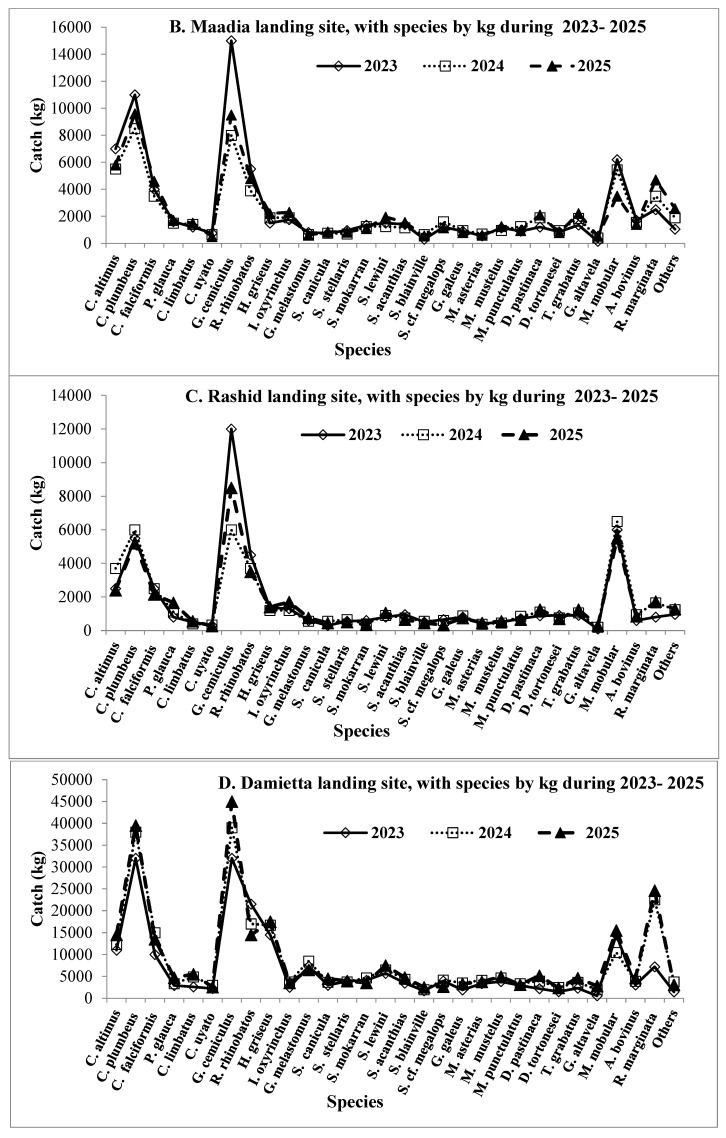

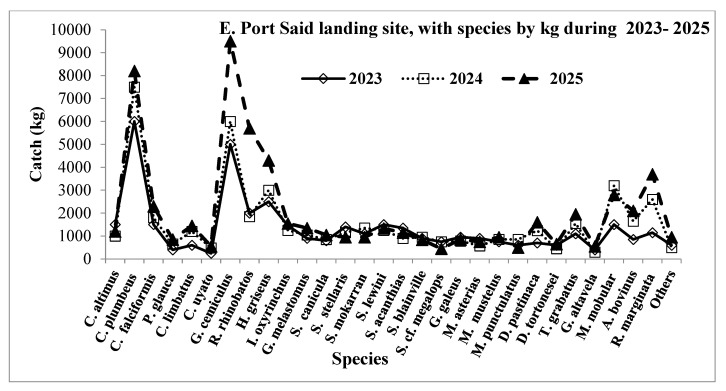
Regional species composition of Major commercial elasmobranchs according to major landing sites along the Egyptian Mediterranean coast: (**A**) Alexandria, (**B**) Maadia, (**C**) Rashid, (**D**) Damietta, and (**E**) Port Said during 2023–2025.

Species composition of major landed elasmobranchs is varied based on region/landing site and are presented in Table 3 and Figure 8. From the table, Damietta and Alexandria still ranked the first landing on the bases of species composition. The primary species were *C. plumbeus* reported 32.000; 38,000 and 39.500 kg in Damietta during 2023, 2024 and 2025 respectively. *C. falciformis* reported 10.000; 15,000 and 13.500 kg in Damietta during 2023, 2024 and 2025 respectively. *G. cemiculus* reported 32.000; 39,000 and 45,500 kg in Damietta during 2023, 2024 and 2025 respectively.

Generally, the species variation during 2023–2025 still located in the highest ranks in Damietta, followed by Alexandria, while Port Said retained the lowest site of landings. Figure 8 represented regional species composition by percentage and catch per kg respectively. From these figures it was noticed that the beaks were observed for species *C. plumbeus*, *G. cemiculus*, *M. mobular* and *R. marginata* during 2023–2025. The beaks of *C. plumbeus*, *G. cemiculus*, and *Rhinoptera marginata* (Geoffroy-Saint-Hilair, 1817) were elevated during 2025 with obvious elevation of *R. marginata* during 2025 in Alexandria and Damietta. For *M. mobular*, its elevated beaks were observed in Rashid followed by Maadia in matching with the high activities of purse seiners.

#### 3.3.2. Fisheries Status and Structure

**Figure 9:**
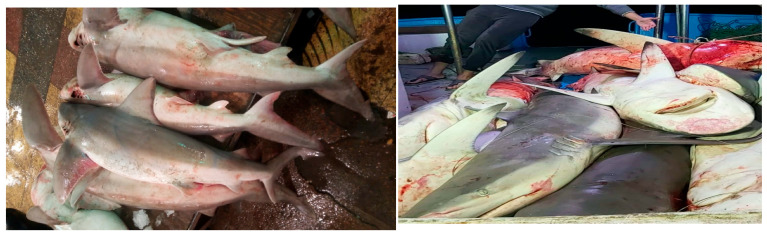

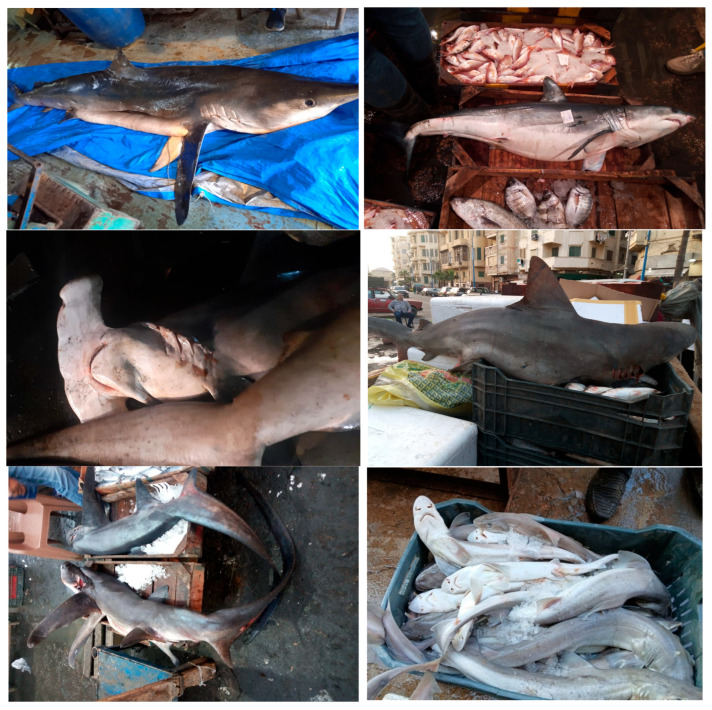

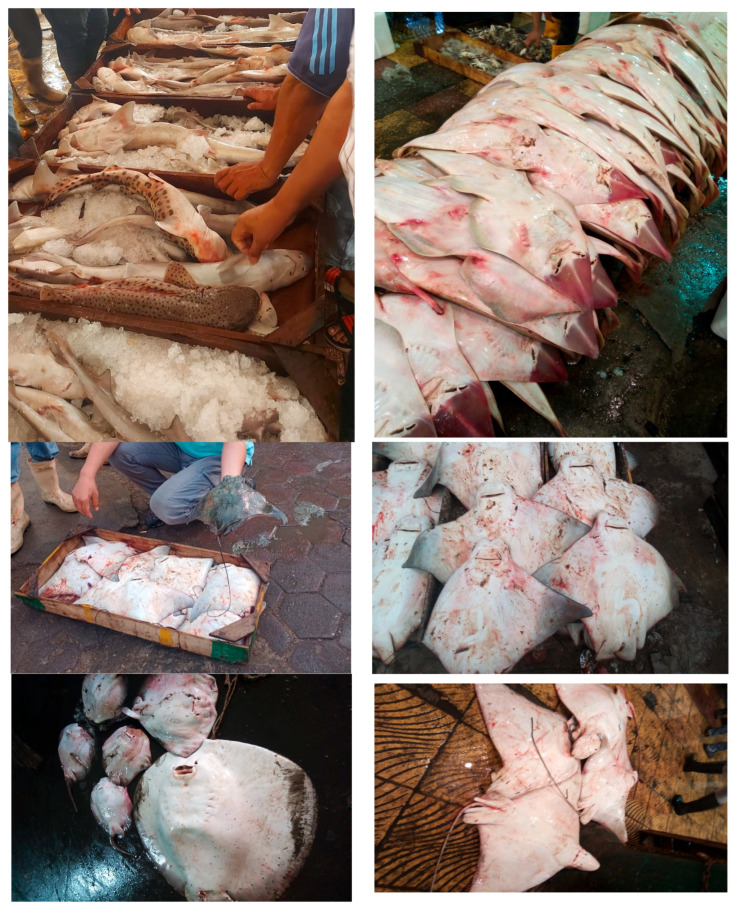
Some of the common elasmobranchs regularly caught as bycatch from various types of fishing gear, and occasionally targeted, are shown, at landing sites along the Egyptian Mediterranean coast (adults and small-sized specimens).

**Figure 10:**
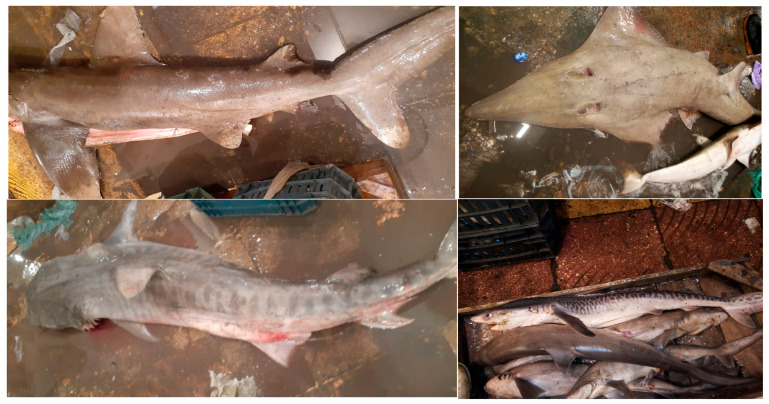
Some of the red Sea elasmobranchs caught from the Suez Gulf and Hurghada and transformed to landing sites (Alexandria and Damietta), with preferable Alexandria as suitable market along the Egyptian Mediterranean coast (Note: many persons thought they caught from Mediterranean Sea as they landed).

The fisheries status and exploitation of elasmobranchs/cartilaginous species along the Egyptian coast have been investigated to understand their dynamics and catchability during the years 2023–2025. Most landed elasmobranchs were caught as bycatch from various types of fishing gear throughout the year. Otherwise, sometimes, particularly during spring and summer, some boats target some species such as *C. plumbeus*, *C. falciformis*, *H. griseus* and *G. cemiculus.* The common regularly landed elasmobranchs (bycatch and targets) included the *C. plumbeus*, *C. altimus*, *C. falciformis*, *I. oxyrinchus*, *H. griseus*, dog sharks (various species), and bull ray *Aetomylaeus bovinus* (Geoffroy St. Hilaire, 1817), while other small-sized species of less importance have less consumer acceptance in comparison to the species occasionally fished throughout the year. The major species that are fished in various quantities and presented in markets and landing sites (Figure 9) reveal the present situation of shark exploitation. Among the fisheries situation of elasmobranchs and landing, several species and quantities had been transformed from the red Sea and Suez gulf (Figure 10).

#### 3.3.3. Length Range and Length Variations

The length range and variations were taken for major landed elasmobranchs as shown in Table 2 prevoiusly. From the table, the total length (TL) of the common sandbar shark *C. plumbeus* showed a wide range, from 75 to 320 TL (cm), while the TL range of *C. falciformis* was 60–210 cm. These are semi-demersal sharks that sometimes inhabit the pelagic layers. The pure pelagic sharks, such as the shortfin mako *Isurus oxyrinchus*, was reported with a wide length range of 35–200 cm (TL), including small-sized specimens collected from inshore water mostly off Alexandria and in Zones C and D, usually from March to June. On the other hand, large specimens were collected from the open water in different zones. The blue shark *P. glauca* had a TL range of 120–280 cm. Regarding small demersal sharks such as dog sharks and catsharks, they were found in a normal length range, starting from 33–56 TL (cm) for *G. melastomus* to 75–125 cm TL for *Scyliorhinus canicula* (Linnaeus, 1758). The smooth-hound, *Mustelus mustelus* (Linnaeus, 1758), representing the common small shark, showed a length range of 43–120 cm (TL). The common deeper shark *H. griseus* showed a length range of 136–380 cm (TL).

Other length keys were obtained for the guitar shark, showing a wide range of 35–200 cm TL for the blackchin guitarfish *G. cemiculus*, and 30 cm to 120 for *R. rhinobatos*. Regarding rays, Table 2 illustrates the normal length ranges for some species, while small-sized specimens were obtained for the Lusitanian cownose ray *R. marginata* (29–140 cm WL); the small-sized specimens were caught by trawl and gillnets from the inshore area during March to June mostly from Zones C and D. The occasional/rare species were measured in few individuals such as the angular rough shark *O. centrina* ((35 cm and 3 kg) and angle shark *Squatina aculeata* (85–120 cm and 7–10 kg). For the newly recorded/updated species, the length range obtained for the basking shark *C. maximus* was 3.5–6 m TL, and, for the kitefin shark *D. licha*., the length ranged from 85–110 cm TL. From the present results, it was noticed that many species exhibited a wide range of lengths, including small-sized species.

#### 3.3.4. Gear Variations

The fishing gear significantly influenced the catchability of elasmobranchs, with the species-specific variations summarized in Table 4. The table presents the percentage of each species captured by different gear types. Overall, trawl nets ranked first in catching elasmobranchs, followed by gillnets, and then longlines. Purse seines and beach seines contributed mainly to incidental and seasonal catches, with beach seines having the greatest impact on small-sized elasmobranchs during the nursery season. Trawl nets and purse seines predominantly captured elasmobranchs as bycatch, whereas gillnets and longlines targeted them both directly and incidentally.

Regarding species variation, gillnets ranked first for catching *C. altimus* (51.5%), *C. plumbeus* (53.5%), *C. falciformis* (57%), *C. limbatus* (46%), *G. cemiculus* (45%), and *R. rhinobatos* (55%), followed by longlines as the second most effective gear. The table also indicates that trawlers were the primary gear for capturing demersal species, particularly dogfish and catsharks. Large rays were frequently caught by bottom and deep trawlers, including *Dasyatis tortonesei* (Capapé, 1975) (60% and 58%), *Taeniurops grabatus* (Geoffroy Saint-Hilaire, 1817) (57.5%), *G. altavela* (70%), *A. bovinus* (45%), and *R. marginata* (39%). In contrast, large pelagic sharks such as *P. glauca* and *I. oxyrinchus* were most commonly caught by longliners, especially drifting longlines, accounting for 50% and 45% of their catches, respectively.

#### 3.3.5. Conservation Indicators and Challenges (Measures and Awareness)

The present survey of cartilaginous species across the studied zones provides a valuable key reference for future conservation planning. In Zone C (off the Delta region, particularly near Alexandria), trawl nets are the primary fishing gear used to capture rays and skates, with sharks occurring occasionally as bycatch. Longlines and hook gear are also employed, targeting several shark species, especially the shortfin mako and silky shark. Notably, juvenile shortfin mako individuals (approximately 35 cm TL; 2.5 kg) were recorded between March and May, likely associated with nearshore foraging on sardines and other small pelagic species. In contrast, adult individuals were more frequently captured during June and July, coinciding with the migration of tuna and sardine schools in both inshore and offshore waters.

In Zone D, the blackchin guitarfish was the most commonly captured species, particularly from May to October, when larger individuals were prevalent. Both small (up to 30 cm) and large specimens (up to 2 m) were observed, with juveniles inhabiting shallow nearshore waters (around 1 m depth) and adults occurring at depths of 6–8 m.

Additionally, schools of small silky sharks were observed in November at depths of 6–9 m close to the shoreline. These patterns suggest that Zone D functions as a potential hotspot for certain elasmobranch species. Zone A, which serves as a border area, experiences limited fishing pressure due to political constraints. However, the adjacent shallow Delta region supports high seasonal abundances of sharks, including sandbar, dusky, and silky sharks, particularly during spring and summer, when adults migrate from offshore to nearshore habitats. This zone also contains scattered habitats suitable for guitarfish. Furthermore, it serves as an important migratory corridor for large pelagic rays, particularly the spinetail devil ray (*M. mobular*), which moves through open waters from west to east.

Field observations also documented localized aggregations of shark species, predominantly sandbar sharks, in nearshore sandy habitats during April, especially in Zone B (adjacent to Zone A) and Zone D. These aggregations increase vulnerability to capture, particularly by longline fisheries. The same criteria were observed for large rays mostly *R. marginata* captured in huge amounts by trawlers in zones B and D during April. It’s very small individuals (29 cm WL), were recorded in trawl catches between May and early June, as well as similarly small specimens of *T. grabata* during October and November. Pregnant females of *I. oxyrinchus* were observed in catches during December. These findings, together with landing data, are illustrated in Figure 9. Overall, elasmobranchs in the study area are predominantly captured as bycatch under irregular fishing activity, with only a limited number of fishers intentionally targeting certain species, mainly sandbar sharks and guitarfish, during specific seasons using gillnets and longlines. These observations highlight the need for improved handling practices and increased awareness to support the effective conservation of elasmobranch populations.

## 4. Discussion

This comprehensive study aims to fill a knowledge gap concerning the overall situation of elasmobranchs along the entire Egyptian Mediterranean coast, focusing on their species diversity, distribution, and fisheries status, including the estimated catchability and gear variations. In the present findings, the total reported species of elasmobranchs amount to 69 species, including 2 chimaeras, 41 sharks, and 26 rays and skates, from shallow, open, and deeper waters. These findings are considered to show the recent updated elasmobranch diversity along the Egyptian Mediterranean coast. This is because the previous reports and studies were focused on specific areas or certain groups of elasmobranchs [14,15,16,17,18,25].

Bradai et al. [30] have mentioned 86 species of elasmobranchs in the Mediterranean Sea (49 sharks from 17 families and 37 batoid species from 9 families), which is higher than the results reported in the present study. According to Bradai et al. [30], the variation in the identification of elasmobranch species between the eastern and western Mediterranean basins may be attributed to ecological differences. This current disparity can also be attributed to the lack of reports on some species in Egypt, especially the deeper-water sharks, considering that deep-sea exploration only commenced in 2009 and has been documented increasingly in subsequent studies from 2011 [19,20,21]. Moreover, the Delta region in Egypt may play a role in fishing activity and introduce a geological barrier for some species coming from the west, leading to a specific distribution of elasmobranchs. Otherwise, the limited scientific attention to elasmobranchs, possibly due to some factors such as their sizes and the need for huge efforts and budgets, and recently, due to restrictions on their fishing and trade by the Egyptian laws. These have led to the decline in targeting these species.

In accordance with Bradai et al. [30]’s criteria, Buencuerpo et al. [31] have suggested that the differences in food variability between western Mediterranean and eastern Atlantic waters influence the variations in diversity between these areas. According to Dulvy et al. [26], the diversity of chondrichthyans was the greatest in the western Mediterranean Basin, particularly in the coastal waters of the North African countries. Serena [4] also reported that the distribution of the Mediterranean elasmobranchs is not homogeneous: the western part is of higher trophic quality compared with the eastern part, which supports this assumption, as the eastern Mediterranean basin is regarded as one of the most oligotrophic regions of the world’s oceans.

Ferretti et al. [32] reported a significant decline in large predatory sharks in the Mediterranean Sea, with a reduction between 96% and 99% compared to the past century. These findings emphasize the ecological challenges faced by elasmobranchs in the Mediterranean, particularly in the eastern basin, due to factors such as the limited nutrient availability, and reduced abundance of living resources.

On the other hand, the current diversity showed that the genus Centrophorus is represented by a single species, *Centrophorus uyato* (Rafinesque, 1810), that was reported in the southeastern Mediterranean, in contrast to the recent work of Serena et al. [33], who reported the presence of two species, *Centrophorus granulosus* and *C. uyato* in Mediterranean Sea. Another study proposed a new description of the species by establishing a neotype for *C. uyato*, as molecular approaches demonstrated the presence of a unique mitochondrial clade within the Mediterranean Sea specimens [34]. This highlights the need for scientists to carefully revise such similarities to confirm whether these are a single species or two separate species.

Further, the present study recorded the basking shark *Cetorhinus maximus* (Gunnerus, 1765) from the coastal zone off Alexandria (captured by gillnets) and off Damietta (captured by trawls) during 2023 and 2024 repectively. This species had never been reported from the Egyptian coast before, and it is considered as a migratory species. The presence of this shark is mainly recorded by incidental catches in trammel nets or in other types of fishing gear which are frequently used in coastal waters in different countries [35,36]. Serena et al. [37] reported that the occurrence of *C. maximus* appears to be confined to the western and central sectors of the Mediterranean Sea, while, in the northwestern part of the Mediterranean, it can be considered to be frequently caught mainly in the spring. Today, the only information about *C. maximus* in the Mediterranean Basin comes from occasional sightings (42%) and incidental catches with trammel nets (15% of the total 323 records analyzed).

The factors explaining the lack of data about the presence of basking sharks in the eastern Mediterranean area could be related to the biological, chemical, and physical characteristics of this area: high-water temperatures and low chlorophyll concentration throughout the year [38]. Few records of such shark sightings in this area correspond to small coastal zones, where the chlorophyll concentration was a little bit higher (Israel, Turkey, and Tunisia) [38]. In addition to its delaying/occasional occurrence in the eastern part such as Egypt, it may face the shallower delta region and may pass via the open water towards Lebanon and turn to Turkey. Therefore, its entrance to the Egyptian coastline may be happened in delayed arrival or incidental reports, particularly in small-sized specimens such as the presented basking shark. This criterion was supported by Farrag et al. [17] for marine mammals in Egypt, which have been sighted along the coast of the Delta during its migration from west to east as a result of climate change.

Limited attention had been given to the fishery of deep-sea sharks [19,20,21], with no reports of the kitefin shark/seal shark (*D. licha*) due to the confusion in its identification with other sharks. The present study has added *D. lichia* to the region’s shark diversity; it was caught by bottom trawlers at depths of more than 350 m. Other updated shark species included the smalltooth sand tiger shark, which was caught as a rare individual from the border area off Egypt near the Libyan Coast. The opposite border areas such as Al-Arish near Palestine faced some restrictions on fishing operations, which lead to low occurring of elasmobranchs reporting.

The present study highlights the presence of significant large pelagic sharks such as blue sharks (*P. glauca*) and shortfin mako (*I. oxyrinchus*), mostly associated with the tuna and sardine seasons. Blue sharks were observed in the open water during the operations of longliners targeting albacore tuna and other tuna species, while the shortfin mako was reported along the coast, mostly during the spring and summer, in open and shallow waters. In the southeastern Mediterranean, the distribution of blue sharks was found to be linked to environmental cues, including ambient temperature, bottom topography, and lunar cycles, with seasonal movements towards coastal areas during the spring, possibly associated with reproduction [39]. The current study reported the blue shark from different locations along the coast of Egypt, starting from Matrouh to Al-Arish. It was caught mainly by longliners during tuna season mostly off Damietta from May to September. This area includes the largest harbor (Ezbet El-Borg) that has a high number of fishers carrying out the tuna and albacore fishing. It is a pity that the global exploitation of blue sharks might be approaching the maximum sustainable yield levels for trade [40].

Shortfin mako was found along the coast, particularly in the Delta region. This region is characterized by increased prey availability during the spring and summer and serves as an attractive location for shortfin mako to seek their prey such as sardine and others. These sharks were caught mainly by hooks and longlines as small-sized specimens near the shore, while large sizes were usually found in the open water, caught by longliners and purse seiners, while occasionally by gill nets. The presence of small-sized fish reflected the nursery and feeding ground. Unfortunately, no detailed data on the distribution of shortfin mako have been published previously in Egypt. The longfin mako was reported in the present study in very few/rare specimens and was not familiar to the fishers. It was reported previously by Azab et al. [16].

Another large shark in the Mediterranean Basin and worldwide is the great white shark *Carcharodon carcharias* (Linnaeus, 1758). Globally, it is found in warm to cool temperate waters, including the Mediterranean Sea, particularly in the western and central regions [41]. Certain marine areas along the Tunisian and Sicilian coasts [42], as well as in the Aegean Sea [43], have been proposed to be the nursery grounds for the Mediterranean white sharks. The seasonal migration of fish such as tuna has attracted large and old great white shark specimens to be caught in Tunisia [44]. This may indicate the possibility of expanding the reports for the great white shark in the eastern part of the Mediterranean Sea such as off the coast of Libya and Egypt. Over time, the eastern Mediterranean Sea may receive several species from the Atlantic and western parts due to climatic changes that influence the movements of small species.

In fact, this species had been previously reported by Akel and Karachle [10] in the eastern Mediterranean Sea, Egypt; however, this report lacked confirmation. To date, no verified observations have been documented from Egyptian Mediterranean waters. This uncertainty likely stems from misidentification, particularly confusion with the porbeagle shark (*Lamna nasus* (Bonnaterre, 1788)) or large individuals of the shortfin mako (*I. oxyrinchus*), as indicated by communications with fishers, spearfishers, and field survey. While this absence of evidence does not preclude its current or future occurrence, reliable identification based on clear diagnostic features, as well as behavior and size, remains essential.

A comparable situation applies to the tiger shark (*Galeocerdo cuvier* (Péron & Lesueur, 1822)), although in this case the issue relates more to its regional occurrence than to misidentification. During the present survey (spanning more than five years), specimens of tiger sharks were not caught by Egyptian fishers in Mediterranean Sea, or observed by spear fishers, in spite of its presence in the landing sites many times in small and large specimens particularly in Alexandria and Damietta. These landed specimens were excluded from the present assessment, as they had been transported from the Red Sea or the Gulf of Suez for trade rather than captured locally in the Mediterranean Sea. Nevertheless, the potential migration of this species through the Suez Canal into the Mediterranean cannot be ruled out, either currently or in the future. Accordingly, continuous updates of species diversity are necessary, with particular emphasis on accurate documentation of capturing locations.

Similarly, the whale shark *Rhincodon typus* Smith, 1828 is expected to occur along the Egyptian Mediterranean coast, either arriving from the Atlantic Ocean or via the Suez Canal. Upon entering the Mediterranean via Suez Canal, prevailing currents may facilitate its movement eastward along the Egyptian coastline toward Al-Arish and subsequently to Gaza, Palestine. This hypothesis is supported by reports of whale shark sightings along the Gaza coast between 2023 and 2025 [45], suggesting a possible migration route through the Suez Canal and into the eastern Mediterranean. However, no confirmed observations of this species have been reported by Egyptian fishers to date. An alternative possibility is that the whale shark reaches the eastern Mediterranean directly from the Atlantic via open waters, not by passing the shallow Delta region. This scenario is supported by documented occurrences of the whale shark from Türkiye (2021) [46], Spain (2022) [47], and Syria (2025) [48]. Both hypotheses regarding its migration pathways remain plausible and warrant further monitoring and investigation. Nevertheless, the Suez Canal remains the most likely corridor, particularly for species originating from the Red Sea, such as the tiger shark and whale shark. Others among listed species which are considered rare with insufficient data such as *B.* cf. *brevicaudata*, they are still being studied currently and need more further data.

Regarding fisheries and fishing gear catching the cartilaginous species, the current survey illustrated that trawl nets were employed to catch a variety of cartilaginous species, particularly rays and skates in shallower waters, while deepwater trawl nets were used to target deepwater shrimp and also catch deeper sharks, mostly dog sharks. This was in accordance with the findings of Ibrahim et al. [19] and Farrag [20,21]. Gillnets catch some sharks and some rays, in small and large sizes. They are considered important gears for catching large quantities of guitar sharks and sandbar sharks, together with other elasmobranch species. The gillnet feedback was in agreement with Ragheb and Hassan [18] who reported small-sized rays off the shore of Alexandria. Longlines and hooks are primarily used for pelagic and demersal sharks, in large- and small-sized sharks, as well as guitarfish specimens. Due to the prohibition of shark fishing and trading, the capture of elasmobranchs is generally considered fisheries’ bycatch, except for a few fishers along the coast who target specific species, such as the sandbar shark, silky shark, and guitar shark during certain seasons, mostly spring and summer.

This study has, for the first time, highlighted the elasmobranchs’ catchability and species composition on the Egyptian coast to reflect their fisheries and marketing situations in Egypt. The annual statistic book of the General Authority for Fish Resources Development (GAFRD) [49] in Egypt has estimated the overall catch of elasmobranchs without the species composition. The total estimated catch was 3333 tons during 2011, 2338 tons during 2012, and 881 tons during 2020 for elasmobranchs along the Egyptian Mediterranean coast [49]. In 2021, the catch was 1050 tons [50]. In the current study, the estimated catches of elasmobranchs from the major landing sites showed lower values than those reported by the GFARD. This may be due to the LERPDA (Lake Fisheries Resources Protection and Development Authority) covering other minor areas. Recently, the fishing of elasmobranchs, particularly sharks, was prohibited due to restrictions in the Egyptian laws which led to a decline in the landed species.

Regarding the catch variations according to the landing sites, the present results revealed that the highest landing site during 2023 was Damietta (208.920 kg; 38.59%) followed by 166,750 kg (30.80%) for the Alexandria landing, while the lowest catch was estimated at Port Said at 40,000 kg (7.39%). The same trend has been estimated during 2024 and 2025, where the highest landing site was Damietta, which constituted 258,725 kg (42.30%) and 274,350 kg (41.20%), followed by 192,195 kg (31.42%) and 210,900 kg (31.68%) for Alexandria during 2024 and 2025, respectively, while the lowest catch was estimated at Port Said at 47,530 kg (7.77%) and 61,020 (9.16%) during 2024 and 2025, respectively.

For seasonal variation, spring ranked first in elasmobranchs catchability, followed by summer season. This pattern contrasts with the typically higher fishing intensity during summer, which generally results in greater other fish catches. However, the seasonal aggregation of many elasmobranch species for breeding during April and May likely explains the elevated catchability observed in spring. Variation during the other seasons may be influenced by differences in fishing activity (going on or stopping due to various circumstances and weather), and the seasonal occurrence of species.

According to the GAFRD, Egypt, the highest number and various fishing vessels were reported in the Damietta region. This may be the reason for its highest rank in terms of its catch. Alexandria is considered the best suitable landing site for elasmobranchs from various fishing gears working in the western part of the Egyptian coast, it is also considered the primary market for elasmobranchs along the coast as well as it receives additional quantities from other areas such as the Red Sea and also from the out Egypt area. This may cause some misidentification in species diversity or even in estimation of various catchability for non-experienced scientists. In the Mediterranean Sea, the elasmobranch catches represent only 1.15 percent of the total landings (FAO Statistics 1980–2015), the major countries fishing elasmobranchs within the Mediterranean were Libya, Tunisia, Italy, and Turkey, and, between 1980 and 2008, they registered a dramatic decrease in terms of their catch [51]. Unfortunately, the catches show a decreasing trend: 26,000 tons in 1983–1984 and 14,000 in 2015. A decline in cartilaginous species landings has been observed while the fishing effort has generally increased, which led to overexploitation and extra bycatch [52], as well as negatively influencing the fishery of another marine megafauna. Besides fishery activities, Mediterranean elasmobranch populations are also affected by pollution and habitat degradation, resulting in drastic population declines [32].

As mentioned before, Egyptian laws prohibit the fishery of sharks; therefore, various types of fishing gear catch elasmobranchs throughout the year or seasonally as bycatch. This agrees with the findings of Bradai et al. [30]. In the present study, gillnets participated in catching common species, such as the guitar shark and sandbar shark, as well as small-sized specimens in nursery grounds. These findings agreed with Costantini et al. [53] and Echwikhi et al. [54], who reported that gillnet fisheries targeting sharks in the Mediterranean Sea were generally seasonal and local. Other types of fishing gear such as longlines were among the important gear for catching both pelagic and demersal sharks and rays. This kind of activity was reported in Tunisia and Libya, which are known for catching sandbar *C. plumbeus* with pelagic longlines (15.22 individuals/1000 hooks) through July–October, and during 2007–2008 [9], and guitarfishes along the Libyan coasts using bottom and pelagic longlines [55]. This indicates that these types of fishing gear, particularly in small-scale fisheries, may negatively influence marine vertebrates, causing the mortality of elasmobranchs and other megafauna. This finding agrees with Soykan et al. [56] and Moore et al. [57]. Similarly, Bradai et al. [30] confirmed that the small-scale fisheries in the Mediterranean Sea introduced a significant source of mortality for the early-life stages of elasmobranch species while operating mainly in nursery areas.

The current study showed that the trawling operations targeting shrimp and other bony fishes in both shallow and deeper water also capture elasmobranchs as bycatch, which was in agreement with previous studies in Egypt [18,20,21,22]. Other studies in different countries showed high numbers of elasmobranch species among trawling catches: 62 species in Greece, 62 species in Catalonia, 74 species in Italian waters, 31 species in the Gulf of Gabès, and 20 species in Iskenderun Bay [58,59]. The variation between the present results and those of other countries, particularly the western and central areas of the Mediterranean Sea, may be attributed to the limited extent of deeper operations with the presence of a shallow habitat topography, such as the Delta region in Egypt, extending over long distances. As mentioned before, such topography acts as a barrier for some species and inhibits deepwater operations, which typically host a greater diversity of elasmobranchs.

The species composition revealed that the highest catches were estimated for the blackchin guitarfish *G. cemiculus* at 92,000 kg (16.99%), 89,500 kg (14.63%), and 109,100 kg (16.39%) during 2023 to 2025, respectively, followed by *C. plumbeus*, which constituted 76,000 kg (14.04%), 88,000 kg (14.39%), and 95,500 kg (14.34%) during 2023, 2024, and 2025, respectively. The large pelagic ray *M. mobular* had a catch of 36,000 kg with a percentage of 6.65% during 2023, and 34,350 kg (5.58%) during 2024 and 32,900 kg (4.94%) during 2025. The present findings reflect a picture of the major landed and exploited species that should be addressed in future further management and conservation.

The length range of species could be used as a key and measure for management. The guitar shark *G. cemiculus* and sandbar shark *C. plumbeus* showed wide ranges of 35–220 cm TL and 75–320 cm TL, respectively. These wide ranges suggest that these populations can sustain exploitation. The shortfin mako showed a length range of 35–200 cm TL, including small-sized specimens. Moreover, large ray species like the Lusitanian cownose ray *R. marginata* showed small-sized specimens in the range of 29–140 cm WL. The length range of most measured species was slightly similar to that reported by Ahmed et al. [60] for *M. mustelus* from Egypt, Wahyudin et al. [61] for *C. falciformis*, and *Sphyrna lewini* (Griffith & Smith, 1834) from Indonesia. Tsakiris and Dimarchopoulou [62] reported the length ranges of 46 sharks and rays from different countries in the Mediterranean Sea. They had smaller ranges than the present results for most estimated species. This reflects some healthy aspects of elasmobranch populations in the area studied. Nevertheless, the present wide range of species may also suggest an absence of awareness during fishing activity in both targets and bycatch, which signals the need for conservation measures for small-sized specimens, particularly for the guitar shark, the shortfin mako, and large ray *R. marginata*. Moreover, the small-sized shortfin mako caught by longline fishing during the late winter–spring in Zones C and D, and the blackchin guitarfish sharks caught in various zones, with a high intensity in Zone C, during the spring–summer seasons indicate that these zones may be nursery grounds for these species, as well as other demersal rays and sharks. Additionally, during April and May, a lot of sharks were aggregated near the shore in large quantities: these criteria were observed for various species, mostly *C. plumbeus*, which is seen in Zone B and the adjacent Zones A and D, as it favors sandy and calm conditions. This exceeds the need for attention regarding this time for aggregation and more control.

The decline in elasmobranch species in the Mediterranean was recognized by the IUCN (International Union for Conservation of Nature) [26] and confirmed by fishers [63]. Spatiotemporal analyses of large shark abundances in the Mediterranean Sea showed that the population status ranged between overexploited and locally depleted [32]. Similarly, this was reported for *Squatina squatina* (Linnaeus, 1758) [64]. In the present results, the Squatina spp. were observed in few numbers, while other species fluctuated between rare, threatened, and exploited. Some of these species agreed with the IUCN list classification, while other species did not reach the critical point, confirming local variations in the population status.

The expected decline in sharks in ecosystems can lead to indirect alterations in the predating pressure on various fish species. Several authorities encourage the evaluation and sustenance of elasmobranchs through conventions, taking into consideration the fact that a greater part of large shark species is classified as threatened, vulnerable, and endangered [65,66]. Conservation measures for many species have been implemented to date in the Mediterranean (except for *C. carcharias*, *C. maximus* under European Community (EC) Regulation 407/2009 [67], and the general shark-finning ban under EC Regulation 1185/2003) [68]). The alarming status of Mediterranean shark species has populated the literature [69,70,71], providing sufficient and conclusive scientific advice for decision makers, as well as guiding rules for effective conservation and management. Large pelagic fish in the Mediterranean are managed by two major organizations: (i) the General Fisheries Commission of the Mediterranean (GFCM), and (ii) the International Commission for the Conservation of Atlantic Tunas (ICCAT). All organizations collaborate on management recommendations and promote data exchange between nations. In Egypt, the LFRPDA and the Ministry of Environment have released some laws during 2012 (Decree 444) to prevent the fishing and trading of sharks for conservation purposes [72]. However, fishing guidelines for elasmobranchs must be enforced in relation to their percentage in the bycatch in order to get a clear picture of their exploitation. Finally, there is a need to increase fishermen’s awareness of prohibited species and educate them on size limits and season restrictions.

## 5. Conclusions

This study provides a comprehensive analysis and valuable insights into the updated diversity and distribution of elasmobranchs along the Egyptian Mediterranean coast, thereby establishing a foundation for future research and conservation efforts. It also presents, for the first time, detailed information on catch composition and species distribution, with reference to landing sites and market dynamics. Elasmobranchs primarily sharks, large rays, and guitarfish—continue to be exploited through both legal and illegal practices, despite the existing ban on shark trading in Egypt. The findings of this investigation highlight several key recommendations. A complete prohibition of elasmobranch fishing may not be the most effective approach; instead, regulated fishing with restrictions on timing and gear is suggested. It is also important to distinguish between elasmobranch populations situation in the Red Sea and those in the Mediterranean Sea. Enhanced monitoring and stricter control measures are particularly needed during April and May, as well as within Zone D as hot spot area.

Regular and systematic monitoring of species diversity is essential to track current trends and anticipate future changes, including potential species migration from the Atlantic Ocean and the Red Sea through the Suez Canal. Fishing gear should be re-evaluated, especially gillnets and trammel nets to reduce bycatch and allow non-target species to escape. Additionally, the use of smaller hook sizes in longline fisheries is recommended. Raising awareness among fishers is crucial, particularly regarding the safe release of non-target, juvenile, and pregnant individuals. Fishing activities in nearshore and shallow waters should be limited, especially for purse seiners, trawlers, gillnets, and beach seines, to protect aggregations of species and conserve the young sharks and rays. Rather than a total ban, implementing a regulated list of permitted species could support both conservation goals and the livelihoods of fishers. The above points considered a challenge facing bycatch and little targets of elasmobranchs, which led to the need for control at landing sites during specific seasons to avoid the capture of small-sized specimens of rays and sharks and certain endangered species.

## Figures and Tables

**Table 1:** Diversity, distribution, gears, depth and fisheries rank status of the cartilaginous fish species reported along the Egyptian Mediterranean coast. A–E are zones of distribution: A. area at the eastern border of Egypt from the side of Egypt from Al-Arish to Port Said; B. area east of the Delta and extending slightly from Port Said to after Rashid; C. area located off the Delta region to Alexandria; D. area from Alexandria to Marsa Matrouh westward; and E. area located in the western part of Egypt to the Sallum region at the border with Libya (IUCN status by Dulvy, et al. [26]). (* indicates species reported in this study, ++: refers to occurrence in zone, ---: refers to the absent in zone, while ++* indicates the presence of species in the present study including confirmation of previous report, expanding in other area and new records, D.D; Data-deficient and None refers to the absence of data). Note: Disappeared species may occur in another study and further investigations. cf; Need more confirmation.

Family	Species	Common Names	Citation	A	B	C	D	E	Fisheries Gears/Depth	Status Egypt/IUCN
**Chimaeras**										
Chimaeridae	*Hydrolagus mirabilis* (Collett, 1904)	Large-eyed rabbitfish	Farrag [20]	---	++	++	++	---	Deepwater trawler (>350 m depth)	Data deficient/ Data deficient
	*Chimaera monstrosa* (Linnaeus, 1758)	Rabbitfish	Farrag [20]	---	++	---	++	---	Deepwater trawler (>350 m depth)	Data-deficient/ Near threatened
**Sharks**										
*Alopiidae*	*Alopias superciliosus* (Lowe, 1841)	Bigeye thresher	Farrag [15]; Present *	---	++*	++	++*	---	Drifting longlines, hooks, purse seiners (surface open water)	D.D./Endangered
	*Alopias vulpinus* (Bonnaterre, 1788)	Common thresher	Present *	---	---	---	---	++*	Drifting Longlines (surface open water)	Rare/Vulnerable (Need confirmation
Carcharhinidae	*Carcharhinus altimus* (Springer, 1950)	Bignose shark	Moftah et al. [14]; Present *	++*	++*	++	++*	---	B. longline/D. longline/gillnets (20–140 m depth)	Exploited/D.D.
	*Carcharhinus brachyurus* (Günther, 1870)	Copper shark	Azab et al. [16]; Present *	++*	++*	++*	---	---	Longline/B. trawl (25 m to 50 m depth)	D.D./D.D.
	*Carcharhinus brevipinna* (Valenciennes, 1839)	Spinner shark	Azab et al. [16]; Present *	---	++*	++*	++*	---	Longline/gillnets (8 m 50 m depth)	Exploited/ Not applicable
	*Carcharhinus falciformis* (Bibron, 1839)	Silky shark	Present *	++*	++	++*	++*	++*	B. longline/D. longline/gillnets (3 m to 140 m depth)	Exploited/None
	*Carcharhinus limbatus* (Valenciennes 1839)	Blacktip shark	Present *	---	++*	++	++*	++*	Longline/gillnets (10 m to 50 m depth)	Exploited/D.D.
	*Carcharhinus obscurus* (Lesueur, 1818)	Dusky shark	Present *	++*	++*	++	++*	---	Longline/gillnets (1 m to 240 m depth)	D.D./D.D.
	*Carcharhinus plumbeus* (Nardo, 1827)	Sandbar shark	Moftah et al. [14]; Present *	++*	++*	++	++	++*	B. longline/D. longline/gillnets/lines/trawl (8 m to 50 m depth)	Exploited/ Endangered
	*Prionace glauca* (Linnaeus, 1758)	Blue shark	Present *	++*	++*	++	++*	---	D. longline/purse seiners (surface open water) (1 m to 170 m depth)	D.D./ Critically endangered
Centrophoridae	*Centrophorus uyato* (Rafinesque, 1810)	Little gulper shark	Farrag [20]	---	++	++	++	---	Shallow–deeper trawler (up to 250 m depth)	D.D./None
Cetorhinidae	*Cetorhinus maximus* (Gunnerus, 1765)	Basking shark	Present *	++*	---	++*	++*	---	Gillnets/trawl (from 12 m to 70 m depth)	Rare/Endangered
Dalatiidae	*Dalatias licha* (Bonnaterre, 1788)	Kitefin shark/Black shark	Present *	---	++*	---	++*	---	Deepwater trawler (>350 m depth)	D.D./Vulnerable
Echinorhinidae	*Echinorhinus brucus* (Bonnaterre, 1788)	Bramble shark	Ibrahim et al. [19]	---	++	---	++	---	Deepwater trawler **(**>300 m depth)	D.D./Endangered
Etmopteridae	*Etmopterus spinax* (Linnaeus, 1758)	Velvet belly lanternshark	Farrag [20]	---	++	---	++	---	Deepwater trawler/longlines (>250 m depth)	D.D./Least concerned
Hexanchidae	*Hexanchus griseus* (Bonnaterre, 1788)	Bluntnose sixgill shark	Ibrahim et al. [19]; Farrag [20]; Present *	---	++	++*	++	---	Deepwater trawler/B. longlines (>250 m depth); sometimes shallow trawler and gillnets	Exploited/ Least concerned
	*Heptranchias perlo* (Bonnaterre, 1788)	Sharpnose sevengill shark	Farrag [21]	---	++	++	++	---	Deepwater trawler: sometimes B. long lines (>250 m depth)	D.D./D.D.
	*Hexanchus vitulus* Springer & Waller 1969	Bigeyed sixgill shark	Present *	---	---	++*	---	---	Deep trawlers (>250 m depth)	D.D./D.D.
Lamnidae	*Carcharodon carcharias* (Linnaeus, 1758)	Great white shark	Akel and Karachle [10]						Not confirmed	None/ Endangered
	*Isurus oxyrinchus* (Rafinesque, 1810)	Shortfin mako	Present *	++*	++*	++*	++*	++*	D. longlines, purse Seine /Trolling and lines (surface in/offshore) (1 m to 40 m depth	Exploited/ Endangered
	*Isurus paucus* (Guitart, 1966)	Longfin mako	Shaban and El-Tabakh [27]; Present *	---	++*	++	---	---	D. longlines (surface in/offshore)	Rare/D.D.
	*Lamna nasus* (Bonnaterre, 1788)	Porbeagle	Present *	---	++*	++*	++*	---	D. longlines and lines (surface in/offshore) (1 m to 50 m depth)	Rare/Endangered
Odontaspididae	*Odontaspis ferox* (Risso, 1810)	Smalltooth sand tiger shark	Present *	---	---	---	++*	++*	B. longlines/gillnets (up to 50 m depth)	Rare/ Endangered
Oxynotidae	*Oxynotus centrina* (Linnaeus, 1758)	Angular rough shark	Moftah et al. [14]; present *	++*	++*	++	++*	---	Deepwater trawler/B. longlines (up to 300 m depth)	Rare/ Endangered
Pentanchidae	*Galeus melastomus* Rafinesque, 1810	Blackmouth catshark	Ibrahim et al. [19]; Farrag [20]	---	++	++*	++	---	Deepwater trawler (up to 200 m depth)	Exploited/Least concerned
Scyliorhinidae	*Scyliorhinus canicula* (Linnaeus, 1758)	Small-spotted catshark	El-Haweet et al. [28]; Moftah et al. [14]; Présent *	---	---	++	++*	++	B. trawler–B. longliners (up to 200 m depth)	Exploited/Least concerned
	*Scyliorhinus stellaris* (Linnaeus, 1758)	Nursehound	Moftah et al. [14]; Present *	---	---	+++	++*	---	B. trawler–B. longliners (up to 200 m depth)	Exploited/ Near threatened
Sphyrnidae	*Sphyrna mokarran* (Rüppell, 1837)	Great hammerhead	Present *	++*	---	++*	++*	++*	Gillnets/longlines (up to 60 m depth)	Exploited/Not applicable
	*Sphyrna zygaena* (Linnaeus, 1758)	Smooth hammerhead	Present *	++*	---	++*	---	---	Gillnets/longlines (up to 60 m depth)	Exploited/ Endangered
	*Sphyrna lewini* (Griffith & Smith, 1834)	Scalloped hammerhead	Present *	---	++*	---	++*	---	Gillnets/longlines (up to 60 m depth)	Exploited/Not applicable
Somniosidae	*Centroscymnus coelolepis* Barbosa du Bocage & de Brito Capello, 1864	Portuguese dogfish	Present *	---	++*	---	---	---	Deepwater trawler (up to 200 m depth)	Rare/Least concerned
Squalidae	*Squalus acanthias* (Linnaeus, 1758)	Piked dogfish	Moftah et al. [14]; Present *	---	++*	++	++*	---	B. trawl (up to 200 m depth)	Rare/ Endangered
	*Squalus blainville* (Risso, 1827)	Longnose spurdog	Akel and Karachle [10]; present *	---	++*	++*	++*	---	B. trawl (up to 200 m depth)	Exploited/D.D.
	*Squalus* cf. *megalops* (Macleay, 1881)	Shortnose spurdog	Shaban and El-Tabakh [27] Present *	---	++*	++	++*	---	B. trawl (up to 200 m depth)	D.D./D.D.
Squatinidae	*Squatina aculeata* (Cuvier, 1829)	Sawback	Akel and Karachle [10]; Present *	++*	++*	++	++*	---	Trawl/gillnets (7–40 m depth)	Rare/Critically endangered
	*Squatina oculata* (Bonaparte, 1840)	Smoothback angel shark	Moftah et al. [14]; Present *	---	---	++	++*	---	Trawl/gillnets (7–60 m depth)	Rare/Critically endangered
	*Squatina squatina* (Linnaeus, 1758)	Angel shark	Moftah et al. [14]; Present *	---	++	++	++	++*	B. trawl/gillnets (7–40 m depth)	Rare/Critically endangered
Triakidae	*Galeorhinus galeus* (Linnaeus, 1758)	Tope shark	Akel and Karachle [10]; present *	---	++*	++*	++*	++	Shallow–deeper/trawl–longlines (up to 300 m depth)	D.D/Critically endangered
	*Mustelus asterias* (Cloquet, 1819)	Starry smooth hound	Akel and Karachle [10]; Present *	---	++*	++*	++*	---	Shallow–deeper/trawl–longlines (up to 200 m depth)	Exploited/ Vulnerable
	*Mustelus mustelus* (Linnaeus, 1758)	Common smooth hound	Moftah et al. [14]; El-Haweet et al. [28]; Present *	---	++	++*	++*	++	Shallow–deeper/trawl–longlines (up to 200 m depth)	Exploited/ Vulnerable
	*Mustelus punctulatus* (Risso, 1827)	Blackspotted smooth hound	Moftah et al. [14]; Present *	---	++*	++*	++*	---	Shallow–deeper/trawl–longlines (up to 200 m depth)	Exploited/ Vulnerable
**Rays and Skates**										
Dasyatidae	*Bathytoshia lata* (Garman, 1880)	Roughtail stingray	Allam [13]; Present *	---	++*	++	---	---	B. trawler (up to 150 m depth)	D.D./Vulnerable
	*Bathytoshia* cf. *brevicaudata* (Hutton, 1875)	Giant black ray	Present *	---	---	---	++*	---	B. trawl (up to 250 m depth)	D.D./Least concerned
	*Dasyatis pastinaca* (Linnaeus, 1758)	Common stingray	Ibrahim et al. [19]; Farrag et al. [17]; present *	++*	++*	++	++*	++	Shallow–deeper/trawl–longlines (up to 250 m depth)	Exploited/ Vulnerable
	*Dasyatis tortonesei* (Capapé, 1975)	Tortonese’s stingray	Allam [13]; Ragheb and Hasan [18]; present *	---	++*	++	---	---	Shallow–deeper/trawl–gillnet (up to 150 m depth)	D.D./D.D.
	*Pteroplatytrygon violacea* (Bonaparte, 1832)	Blue/Pelagic stingray	Ragheb and Hasan [18]; Present *	---	++*	++	---	---	B. trawl/gillnet nets (up to 80 m depth)	Exploited/ Least concerned
	*Taeniurops grabatus* (Geoffroy Saint-Hilaire, 1817)	Round stingray	Allam [13]; Present *	---	++*	++	++*	---	B. trawl/longlines (up to 200 m depth)	Exploited/ Near threatened
	*Himantura uarnak* (Gmelin, 1789)	Honeycomb stingray	Allam [13]; El-Haweet et al. [28]; present *	---	---	++	++*	++	B. trawl–longlines (up to 80 m depth)	Rare/ Endangered
Gymnuridae	*Gymnura altavela* (Linnaeus, 1758)	Spiny butterfly	Allam [13]; Farrag et al. [17]; Present *	---	++*	++	---	---	B. trawler (25 m to 90 m depth)	Exploited/ Endangered
Mobulidae	*Mobula mobular* (Bonnaterre, 1788)	Spinetail devil ray	Present *	++*	++*	++	++	---	Daytime purse seiners (surface up to 50 m)	Exploited/ Endangered
Myliobatidae	*Myliobatis aquila* (Linnaeus, 1758)	Common eagle ray	El-Haweet, et al. [28]; Present *	---	++*	++*	---	++	B. trawl/lines/seiners (surface to 100 m depth)	Exploited/ Vulnerable
	*Aetomylaeus bovinus* (Geoffroy St. Hilaire, 1817)	Bull ray	Allam [13]; Present *	++*	++*	++	++*	++*	B. trawler/spear/lines (surface to 800 m depth)	Exploited/ Endangered
Rajiidae	*Dipturus oxyrinchus* (Linnaeus, 1758)	Longnose skate	Ibrahim et al. [19]. Farrag [20]	---	++	---	++	---	B. trawl (up to 200 m depth)	D.D./Vulnerable
	*Leucoraja circularis* (Couch, 1838)	Sandy ray	Akel and Karachle [10]; Present *	---	++*	---	---	---	B. trawl (up to 250 m depth)	Rare/Critically endangered
	*Raja asterias* (Delaroche, 1809)	Starry ray	Ibrahim et al. [19]; Farrag [20]	---	++	---	++	---	B. trawl (40 to 200 m depth)	Rare/ Near threatened
	*Rostroraja alba* (Lacepède, 1803)	Bottlenose skate	Ibrahim et al. [19]; Farrag [20]	---	++	---	++	---	B. trawl (100 to 350 m depth)	Exploited/ Endangered
	*Raja clavata* (Linnaeus, 1758)	Thornback ray	Allam [13]; Present *	---	++*	++	++*	---	B. trawler (20 to 150 m depth)	Exploited/ Endangered
	*Raja miraletus* (Linnaeus 1758)	Brown ray	El-Haweet et al. [28]; Farrag et al. [17]	---	++	++	---	++	B. trawl (20 to 150 m depth)	D.D./Least concerned
	*Raja montagui* (Fowler, 1910)	Spotted ray	Akel and Karachle [10]; Present *	---	---	---	++*	---	B. trawl (Up to 80 m depth)	D.D./ Least concerned
	*Raja radula* (Delarochae, 1809)	Rough ray	Allam [13]; El-Haweet et al. [28]; present*	---	++*	++	++*	++	B. trawl (Up to 80 m depth	D.D./ Endangered
Glaucostegidae	*Glaucostegus cemiculus* (Geoffroy St. Hilaire, 1817)	Blackchin guitarfish	Allam [13]; present *	---	++*	++	++*	++*	Gillnets/B. trawl, longlines/spear (2 m to 60 m depth)	Exploited/ Endangered
	*Glaucostegus halavi* (Forsskål, 1775)	Halavi guitarfish	Galanidi et al. [29]; Present *	---	---	++	++*	---	Gillnets/B.trawl, longlines/spear (6 m to 40 m depth)	D.D./None
Rhinobatidae	*Rhinobatos rhinobatos* (Linnaeus, 1758)	Common guitarfish	Allam [13]; Present *	---	++*	++	++*	---	Gillnets/B. trawl, longlines/spear (6 m to 40 m depth)	Exploited/ Endangered
Rhinopteridae	*Rhinoptera marginata* (Geoffroy-Saint-Hilair, 1817)	Lusitanian cow ray	Ragheb and Hasan [18]; Present *	---	++*	++	++*	---	Gillnets/B. trawl/longlines (6 m to 150 m depth)	Exploited/D.D.
Torpedinidae	*Torpedo marmorata* (Risso, 1810)	Marbled electric ray	Allam [13]; Farrag et al. [17]	---	++	++	---	---	B. trawl (6 m to 150 m depth)	Rare/Least concerned
	*Tetronarce nobiliana* (Bonaparte, 1835)	Atlantic new British torpedo	Akel and Karachle [10]; Present *	---	---	++	++*	---	B. trawl (up to 250 m depth)	Rare/Least concerned
	*Torpedo torpedo* (Linnaeus, 1758)	Common torpedo/Eyed electric ray	Allam [13]; Farrag et al. [17]	---	++	++	---	---	B. trawl (6 m to 150 m depth)	Rare/Least concerned

**Table 2:** Annual species composition of the major elasmobranchs (kg), and its percentage relative to the total catch and length range from all landing sites along the Egyptian Mediterranean coast during 2023–2025. TL: total length, WL: wing length. C. sexes: combined sexes.

Species	Length Range (cm) (C. Sexes)	Total Catch 2023		Total Catch 2024		Total Catch 2025	
		kg	%	kg	%	kg	%
*C. altimus*	70–193 TL	34,000	6.28	36,500	5.968	41,900	6.29
*C. plumbeus*	75–320 TL	76,000	14.04	88,000	14.39	95,500	14.34
*C. falciformis*	60–210 TL	30,000	5.54	33,300	5.45	32,050	4.81
*P. glauca*	120–280 TL	7800	1.44	9000	1.48	11,100	1.67
*C. limbatus*	100–190 TL	7300	1.35	12,400	2.03	12,700	1.91
*C. uyato*	41–91 TL	5850	1.08	6930	1.13	5670	0.85
*G. cemiculus*	35–220 TL	92,000	16.99	89,500	14.63	109,100	16.39
*R. rhinobatos*	30–120 TL	46,000	8.50	35,050	5.73	38,300	5.75
*H. griseus*	136–380 TL	29,600	5.47	35,300	5.77	38,600	5.80
*I. oxyrinchus*	35–200 TL	9200	1.70	10,900	1.78	11,700	1.76
*G. melastomus*	33–56 TL	14,200	2.62	15,950	2.61	13,400	2.01
*S. canicula*	75–125 TL	7500	1.39	8450	1.38	9800	1.47
*S. stellaris*	65–110 TL	9700	1.79	8800	1.44	8580	1.30
*S. mokarran*	200–320 TL	10,400	1.92	11,250	1.84	8100	1.22
*S. lewini*	130–210 TL	16,200	2.99	15,600	2.55	17,130	2.57
*S. acanthias*	70–105 TL	11,300	2.09	10,100	1.65	11,250	1.69
*S. blainville*	70–90 TL	4200	0.78	6050	0.99	6090	0.92
*S.* cf. *megalops*	45–70 TL	8750	1.62	9650	1.58	6430	0.97
*G. galeus*	90–150 TL	6250	1.15	8800	1.44	8140	1.22
*M. asterias*	70–95 TL	7150	1.32	7400	1.21	7650	1.15
*M. mustelus*	43–110 TL	8650	1.60	9890	1.62	10,000	1.50
*M. punctulatus*	85–145 TL	6550	1.21	8175	1.34	7300	1.10
*D. pastinaca*	35–60 WL	7550	1.39	11,940	1.95	14,300	2.15
*D. tortonesei*	45–65 WL	5500	1.02	6460	1.06	6800	1.02
*T. grabatus*	25–120 WL	8500	1.57	12,700	2.08	15,700	2.36
*G. altavela*	50–110 WL	1640	0.30	3450	0.56	5560	0.84
*M. mobular*	120–280 WL	36,000	6.65	34,350	5.62	32,900	4.94
*A. bovinus*	70–160 WL	10,200	1.88	11,450	1.87	13,450	2.02
*R. marginata*	29–140 WL	17,500	3.23	44,100	7.21	55,330	8.31
Others		5960	1.10	10,200	1.67	11,290	1.70
Total (kg)		541,450	100.00	611,640	100.00	665,820	100.00

**Table 3:** Regional composition of the major elasmobranchs (kg) from the major landing sites along the Egyptian coast of the Mediterranean Sea during 2023–2025. SE: Standard Error.

Species	Alexandria			Maadia			Rashid			Damietta			Port said		
	2023	2024	2025	2023	2024	2025	2023	2024	2025	2023	2024	2025	2023	2024	2025
*C. altimus*	12,000	14,000	18,000	7000	5500	5800	2500	3700	2400	11,000	12,300	14,500	1500	1000	1200
*C. plumbeus*	21,500	28,000	33,000	11,000	8500	9600	5500	6000	5200	32,000	38,000	39,500	6000	7500	8200
*C. falciformis*	12,000	10,500	9500	4000	3500	4600	2500	2500	2150	10,000	15,000	13,500	1500	1800	2300
*P. glauca*	2100	2400	2200	1600	1500	1700	800	1100	1650	2900	3350	4700	400	650	850
*C. limbatus*	2400	4500	3700	1200	1400	1500	500	400	550	2600	4900	5500	600	1200	1450
*C. uyato*	2350	2520	1800	750	650	500	300	330	270	2200	2950	2550	250	480	550
*G. cemiculus*	28,000	30,500	36,600	15,000	8000	9500	12,000	6000	8500	32,000	39,000	45,000	5000	6000	9500
*R. rhinobatos*	12,500	8600	9800	5500	3900	4800	4500	3700	3500	21,500	17,000	14,500	2000	1850	5700
*H. griseus*	9750	12,500	13,200	1500	1900	2200	1350	1200	1400	14,500	16,700	17,500	2500	3000	4300
*I. oxyrinchus*	2200	2800	2650	1750	1900	2300	1300	1200	1700	2500	3750	3500	1450	1250	1550
*G. melastomus*	4600	5200	4200	800	650	600	600	550	750	7300	8500	6500	900	1050	1350
*S. canicula*	2700	2650	2950	750	750	850	300	550	450	2950	3650	4500	800	850	1050
*S. stellaris*	2900	2550	2350	950	700	830	550	650	500	3900	3750	3950	1400	1150	950
*S. mokarran*	3250	3600	2200	1350	1250	1100	600	400	350	4100	4650	3500	1100	1350	950
*S. lewini*	6700	5700	5300	1500	1250	1950	800	900	1050	5700	6500	7500	1500	1250	1330
*S. acanthias*	4150	2950	3150	1400	1150	1550	950	750	650	3450	4350	4750	1350	900	1150
*S. blainville*	800	1950	1750	300	650	550	500	550	450	1700	1950	2500	900	950	840
*S.* cf. *megalops*	2200	2550	1850	1200	1600	1150	650	600	330	3950	4150	2650	750	750	450
*G. galeus*	1700	2700	2450	900	950	800	800	870	740	1900	3500	3300	950	780	850
*M. asterias*	1950	1650	2100	550	700	600	400	375	450	3350	4100	3750	900	575	750
*M. mustelus*	2450	2950	2300	1150	960	1250	500	450	550	3800	4675	4950	750	855	950
*M. punctulatus*	1450	1875	2100	900	1250	950	700	850	650	2900	3350	3100	600	850	500
*D. pastinaca*	2600	3750	4200	1200	1900	2100	880	1100	1250	2170	3950	5150	700	1240	1600
*D. tortonesei*	1650	1850	2300	850	950	800	900	760	700	1500	2450	2350	600	450	650
*T. grabatus*	2850	4600	5700	1350	1850	2200	900	1050	1250	2300	3650	4600	1100	1550	1950
*G. altavela*	450	600	1400	150	400	530	90	200	280	550	1950	2800	400	300	550
*M. mobular*	7800	8700	5600	6200	5450	3500	6000	6500	5500	14,500	10,500	15,500	1500	3200	2800
*A. bovinus*	4000	3400	4500	1700	1550	1400	600	950	850	3050	3900	4600	850	1650	2100
*R. marginata*	5850	13,850	20,600	2500	3500	4700	800	1650	1730	7200	22,500	24,600	1150	2600	3700
Others	1900	2800	3450	1050	1900	2570	960	1250	1270	1450	3750	3050	600	500	950
Total	166,750	192,195	210,900	76,050	66,110	72,480	49,730	47,085	47,070	208,920	258,725	274,350	40,000	47,530	61,020
%	30.79	31.42	31.68	14.04	10.81	10.89	9.19	7.70	7.07	38.59	42.30	41.20	7.39	7.77	9.16
Average	5558.33	6406.5	7030	2535	2203.67	2416	1657.67	1569.5	1569	6964	8624.17	9145	1333.33	1584.33	2034
SE	1145.47	1318.65	1628.00	610.20	385.02	439.45	447.53	324.31	338.59	1513.72	1757.08	1928.26	226.61	289.01	403.59

**Table 4:** Percentages of the major estimated elasmobranchs (kg) per species in all landing sites along the Egyptian Mediterranean coast during 2023–2025, with gear percentage by species (%). (B: bottom, D: drifting, Ní: night, DT: Daytime, ---: refers to the absent in zone).

Species	Gear Percentage (%)									
	B.Trawl	Deeper Trawl	D. Longlines	B. Longlines	Ni. Purse Seine	D T. Purse Seine	Beach Seine	Gillnets	Hand Lines	Trolling
*C. altimus*	2	---	16	25	1	1	---	51.5	2	1.5
*C. plumbeus*	0.5	---	22	28	1	1	---	53.5	3	1
*C. falciformis*	0.5	---	25	14	2	0.5	---	57	1	00
*P. glauca*	0.5	---	50	0.5	6	15	---	15	3	10
*C. limbatus*	1	---	19	21	2	4	---	46	3	4
*C. uyato*	25	55	---	15	---	---	---	---	5	---
*G. cemiculus*	14	---	2	28	2	3	5	45	1	---
*R. rhinobatos*	12	---	---	25	1	1	5	55	1	---
*H. griseus*	10	55	---	30	---	---	---	---	5	---
*I. oxyrinchus*	1	---	45	2	5	20	2	3	7	15
*G. melastomus*	35	30	---	15	---	---	---	15	5	---
*S. canicula*	40	35	---	10	---	---	---	10	5	---
*S. stellaris*	35	42	---	17	---	---	---	3	3	---
*S. mokarran*	5	3	2	45	---	---	---	41	3	1
*S. lewini*	8	2	4	40	---	---	---	40	5	1
*S. acanthias*	15	54	---	20	---	---	---	7	4	---
*S. blainville*	28	50	---	15	---	---	---	5	2	---
*S.* cf. *megalops*	15	55	---	26	---	---	---	3	1	---
*G. galeus*	15	40	---	25	---	---	---	4	1	---
*M. asterias*	15	35	---	25	---	---	---	4	1	---
*M. mustelus*	25	40	---	25	---	---	0.5	6	2	--
*M. punctulatus*	15	45	---	20	---	---	---	---	2	---
*D. pastinaca*	40	20	---	20	---	---	5	10	5	---
*D. tortonesei*	40	18	---	25	---	---	2	13	2	---
*T. grabatus*	42.5	15	---	31	---	---	2	8	1.5	---
*G. altavela*	60	10	3	12	---	---	3	11	1	
*M. mobular*	13	---	---	1	15	70	0.5	0.5	--	---
*A. bovinus*	40	5	5	5	2	12	7	23.5	0.5	---
*R. marginata*	36	3	11	5	3	14	8	18	2	---
Others	25	30	2	5	2	3	15	15	1	2

## Data Availability

All data generated or analyzed for this study are included within the article.

## References

[1] Nelson J.S., Grande T.C., Wilson M.V.H. (2016). Fishes of the World.

[2] Roskov Y., Ower G., Orrell T., Nicolson D., Bailly N., Kirk P.M., Bourgoin T., DeWalt R.E., Decock W., Nieukerken E. (2019). Species 2000 & ITIS Catalogue of Life, 25 March 2019.

[3] Coll M., Piroddi C., Steenbeek J., Kaschner K., Lasram F.B.R., Aguzzi J., Ballesteros E., Bianchi C.N., Corbera J., Dailianis T. (2010). The Biodiversity of the Mediterranean Sea: Estimates, Patterns, and Threats. PLoS ONE.

[4] Serena F. (2005). Field Identification Guide to the Sharks and Rays of the Mediterranean and Black Sea.

[5] Iglésias S.P. (2014). Handbook of the Marine Fishes of Europe and Adjacent Waters (A Natural Classification Based on Collection Specimens, with DNA Barcodes and Standardized Photographs)-Volume I (Chondrichthyans and Cyclostomata).

[6] Dulvy N.K., Fowler S.L., Musick J.A., Cavanagh R.D., Kyne P.M., Harrison L.R., Carlson J.K., Davidson L.N.K., Fordham S.V., Francis M.P. (2014). Extinction Risk and Conservation of the World’s Sharks and Rays. eLife.

[7] Otero M., Serena F., Gerovasileiou V., Barone M., Bo M., Arcos J.M., Vulcano A., Xavier J. (2019). Identification Guide of Vulnerable Species Incidentally Caught in Mediterranean Fisheries.

[8] Barone M., Mazzoldi C., Serena F. (2022). Sharks, Rays and Chimaeras in Mediterranean and Black Seas–Key to Identification.

[9] Chuang P.S., Hung T.C., Chang H.A., Huang C.K., Shiao J.C. (2016). The Species and Origin of Shark Fins in Taiwan’s Fishing Ports, Markets, and Customs Detention: A DNA Barcoding Analysis. PLoS ONE.

[10] Akel E.H.K., Karachle P.K. (2017). The Marine Ichthyofauna of Egypt. Egypt. J. Aquat. Biol. Fish..

[11] Mazhar F.M. (1974). The Elasmobranchs of the Mediterranean. IV—The Spiny Dogfish, Squalus Fernandinus. Bull. Inst. Oceanogr. Fish..

[12] Hosny C.F. (1981). Studies on Fishes of Family Triakidae off Alexandria. Master’s Thesis.

[13] Allam S.M. (1989). Revision of Order Hypotremata along the Mediterranean Coast off Alexandria with Special Reference to Family Dasyatidae. Ph.D. Thesis.

[14] Moftah M., Aziz S.H.A., El Ramah S., Favereaux A. (2011). Classification of Sharks in the Egyptian Mediterranean Waters Using Morphological and DNA Barcoding Approaches. PLoS ONE.

[15] Farrag M.M.S. (2017). New Record of the Bigeye Thresher Shark, Alopias Superciliosus Lowe, 1841 (Family: Alopiidae) from the Eastern Mediterranean Sea, Egypt. Int. J. Fish. Aquat. Stud..

[16] Azab A.M., Khalaf-Allah H.M.M., Sarhan M.M.H., El-Tabakh M.A.M. (2019). Carcharhinid Shark Species (Family Carcharhinidae), with Special Reference to the First Records in the Egyptian Mediterranean Waters, Alexandria, Egypt. Egypt. J. Aquat. Biol. Fish..

[17] Farrag M.M.S., Ahmed H.O., Toutou M.M., Eissawi M.M. (2019). Marine Mammals on the Egyptian Mediterranean Coast “Records and Vulnerability”. Int. J. Ecotoxicol. Ecobiol..

[18] Ragheb E., Hasan M.W.A. (2021). First Record of Pteroplatytrygon violacea (Bonaparte, 1832) with Annotation of Cartilaginous Fishes by-Catch by Gill Nets (Egyptian Mediterranean). Egypt. J. Aquat. Res..

[19] Ibrahim M.A., Hasan M.W.A., El-Far A.M.M., Farrag E.-S.F.E., Farrag M.M.S. (2011). Deep Sea Shrimp Resources in the Southeastern Mediterranean Waters of Egypt. Egypt. J. Aquat. Res..

[20] Farrag M.M.S. (2016). Deep-Sea Ichthyofauna from Eastern Mediterranean Sea, Egypt: Update and New Records. Egypt. J. Aquat. Res..

[21] Farrag M.M.S. (2022). An Evaluation of the Deep-Sea Catch in the Mediterranean Sea, Egypt Regarding Pattern of CPUE, Diversity, Sharks, and Discards. Sci. Afr..

[22] Froese R., Pauly D. (2024). FishBase. World Wide Web Electronic Publication. http://www.fishbase.org.

[23] WoRMS Editorial Board World Register of Marine Species. Checklist Dataset. https://www.marinespecies.org/imis.php?dasid=1447&doiid=170.

[24] Akel E.-S.H. (2009). Fisheries of Experimental Purse Seine Net Using Light and Population Dynamics of Sardinella Aurita (Family Clupeidae) East of Alexandria, Egypt. Egypt. J. Aquat. Biol. Fish..

[25] Farrag M.M.S. (2014). Fisheries and Biological Studies on Lessepsian Pufferfish, Lagocephalus sceleratus (Gmelin, 1789) (Family: Tetraodontidae) in the Egyptian Mediterranean Waters. Ph.D. Thesis.

[26] Dulvy N.K., Allen D.J., Ralph G.M., Walls R.H.L. (2016). The Conservation Status of Sharks, Rays and Chimaeras in the Mediterranean Sea.

[27] Shaban W.M., El-Tabakh M.A.M., History A. (2019). New Records, Conservation Status and Pectoral Fin Description of Eight Shark Species in the Egyptian Mediterranean Waters. Egypt. J. Aquat. Biol. Fish..

[28] El-Haweet A., Fishar M.R., Geneid Y., Essam Abdel-Moula E. (2011). Assessment of Fisheries and Marine Biodiversity of Sallum Gulf, Egypt. Int. J. Environ. Sci. Eng..

[29] Galanidi M., Aissi M., Ali M., Bakalem A., Bariche M., Bartolo A.G., Bazairi H., Beqiraj S., Bilecenoglu M., Bitar G. (2023). Validated Inventories of Non-Indigenous Species (NIS) for the Mediterranean Sea as Tools for Regional Policy and Patterns of NIS Spread. Diversity.

[30] Bradai M.N., Saidi B., Enajjar S. (2012). Elasmobranchs of the Mediterranean and Black Sea: Status, Ecology and Biology Bibliographic Analysis.

[31] Buencuerpo V., Rios S., Morón J. (1998). Pelagic Sharks Associated with the Swordfish, Xiphias gladius, Fishery in the Eastern North Atlantic Ocean and the Strait of Gibraltar. Fish. Bull..

[32] Ferretti F., Myers R.A., Serena F., Lotze H.K. (2008). Loss of Large Predatory Sharks from the Mediterranean Sea. Conserv. Biol..

[33] Serena F., Abella A.J., Bargnesi F., Barone M., Colloca F., Ferretti F., Fiorentino F., Jenrette J., Moro S. (2020). Species Diversity, Taxonomy and Distribution of Chondrichthyes in the Mediterranean and Black Sea. Eur. Zool. J..

[34] Veríssimo A., Cotton C.F., Buch R.H., Guallart J., Burgess G.H. (2014). Species Diversity of the Deep-Water Gulper Sharks (Squaliformes: Centrophoridae: Centrophorus) in North Atlantic Waters—Current Status and Taxonomic Issues. Zool. J. Linn. Soc..

[35] Serena F., Vacchi M. (1996). La Presenza Dello Squalo Elefante (Cetorhinus Maximus Gunnerus) Nel Tirreno Settentrionalee Nel Mar Ligure. Biol. Mar. Mediterr..

[36] Francis M.P., Duffy C. (2002). Distribution, Seasonal Abundance and Bycatch of Basking Sharks (Cetorhinus Maximus) in New Zealand, with Observations on Their Winter Habitat. Mar. Biol..

[37] Serena F., Vacchi M., Notarbartolo Di Sciara G., Séret B., Sire J.-Y. (2000). Geographical Distribution and Biological Information on the Basking Shark, Cetorhinus Maximus in the Tyrrhenian and Liguarian Seas. Proceedings of the 3rd European Elasmobranch Association (EEA) Meeting.

[38] Mancusi C., Simona C., Aferonte M., Bradai M.N., Hemida F., Fabrizio S., Soldo A., Vacchi M. (2005). On the presence of Basking Shark (Cetorhinus Maximus) in the Mediterranean Sea. Cybium.

[39] Damalas D., Megalofonou P. (2010). Environmental Effects on Blue Shark (Prionace Glauca) and Oilfish (Ruvettus Pretiosus) Distribution Based on Fishery-Dependent Data from the Eastern Mediterranean Sea. J. Mar. Biol. Assoc. U. K..

[40] Clarke S.C., McAllister M.K., Milner-Gulland E.J., Kirkwood G.P., Michielsens C.G.J., Agnew D.J., Pikitch E.K., Nakano H., Shivji M.S. (2006). Global Estimates of Shark Catches Using Trade Records from Commercial Markets. Ecol. Lett..

[41] Boldrocchi G., Kiszka J., Purkis S., Storai T., Zinzula L., Burkholder D. (2017). Distribution, Ecology, and Status of the White Shark, Carcharodon carcharias, in the Mediterranean Sea. Rev. Fish Biol. Fish..

[42] Bradaï M.N., Saïdi B. (2013). On the Occurrence of the Great White Shark (*Carcharodon carcharias*) in Tunisian Coasts. Rapp. Comm. Int. Mer. Médit..

[43] Kabasakal H.A. (2020). Exploring a possible nursery ground of white shark Carcharodon carcharias in Edremit Bay (northeastern Aegean Sea, Turkey). J. Black Sea/Medit. Environ..

[44] Zaouali J., Rafrafi -Nouira S., Amor O.K., Amor M.M., Capape C. (2020). Capture of a Large Great White Shark, *Carcharodon carcharias* (Lamnidae) from the Tunisian Coast (Central Mediterranean Sea): A Historical and Ichthyological Event. Ann. Ser. Hist. Nat..

[45] Abd Rabou A.N., Aitah E.I., Samara S.K., Alajrami M., Jebril M.A., Dardona Z.W., Harbid I.A.A., Salah J.Y., Awadalah S.M., Saqallah W.M. (2026). First Record of the Globally Endangered Whale Shark (Rhincodon Typus Smith, 1828) in the Mediterranean Waters of the Gaza Strip, Palestine, and Its Consumption Shortly After the Ceasefire Following the Two-Year Israeli War of Genocide (2023-2025). Examines Mar. Biol. Oceanogr..

[46] Turan C., Gürlek M., Ergüden D., Kabasakal H. (2021). A new record for the shark fauna of the mediterranean sea: Whale shark, *Rhincodon typus* (Orectolobiformes: Rhincodontidae). Ann. Ser. Hist. Nat..

[47] Kennedy P.M. (2022). Watch: Unbelievable Scenes of a Whale Shark on the Spanish Coast. https://euroweeklynews.com/2022/12/10/watch-unbelievable-scenes-of-a-whale-shark-on-the-spanish-coast/.

[48] Crimson Publishers (2025). Rare occurrence of the whale shark Rhincodon typus (Smith, 1828) off the Syrian coast (Eastern Mediterranean). Echo International Journal of Marine Biology and Oceanography.

[49] GAFRD (2018). General Authority for Fish Resources Development. Fish Statistics Yearbook.

[50] LFRPDA (Lakes and Fish Resources Protection and Development) (2021). Book Year of Fishery Statistic.

[51] Bradai M.N., Saidi B., Enajjar S. (2018). Overview on Mediterranean Shark’s Fisheries: Impact on the Biodiversity (in Marine Ecology—Biotic and Abiotic Interactions).

[52] Hall M.A., Alversonà D.L., Metuzals K.I. (2000). By-Catch: Problems and Solutions. Mar. Pollut. Bull..

[53] Costantini M., Bernardini M., Cordone P., Guilianini P.G., Orel G. (2000). Observations on Fishery, Feeding Habits and Reproductive Biology of Mustelus mustelus (Chondrichthyes, Triakidae) in Northern Adriatic Sea. Biol. Mar. Mediterr..

[54] Echwikhi K., Saidi B., Bradai M.N., Bouain A. (2013). Preliminary Data on Elasmobranch Gillnet Fishery in the Gulf of Gabès, Tunisia. J. Appl. Ichthyol..

[55] Bradai M.N., Saidi B., Enajjar S., Bouain A. (2006). The Gulf of Gabès: A Spot for the Mediterranean Elasmobranchs. the Proceedings of the Workshop on Mediterranean Cartilaginous Fish with Emphasis on Southern and Eastern Mediterranean.

[56] Soykan C.U., Moore J.E., Ždelis R., Crowder L.B., Safina C., Lewison R.L. (2008). Why Study Bycatch? An Introduction to the Theme Section on Fisheries Bycatch. Endanger. Species Res..

[57] Moore J.E., Cox T.M., Lewison R.L., Read A.J., Bjorkland R., McDonald S.L., Crowder L.B., Aruna E., Ayissi I., Espeut P. (2010). An Interview-Based Approach to Assess Marine Mammal and Sea Turtle Captures in Artisanal Fisheries. Biol. Conserv..

[58] Bertrand J., Gil De Sola L., Papakonstantinou C., Relini G., Souplet A. (2000). Contribution on the Distribution of the Elasmobranchs in the Mediterranean Sea (from the MEDITS Surveys). Biol. Mar. Mediterr..

[59] Hamdaoui B. (2009). Les Élasmobranches dans les Débarquements des Chalutiers au Port de Pêche de Sfax, Golfe de Gabès. Master’s Thesis.

[60] Azab A.M., Khalaf-Allah H.M.M., Mohamed A.M., El-Tabakh M.A.M. (2022). Growth and Mortality Rates for Management of the Common Smooth-Hound Shark, Mustelus mustelus in the Egyptian Mediterranean Waters. Egypt. J. Aquat. Biol. Fish..

[61] Wahyudin I., Kamal M.M., Fahrudin A., Boer M. (2019). Length-Weight Relationship and Reproductive Size of Silky Shark Carcharhinus falciformis and Scalloped Hammerhead Shark Sphyrna lewini Collected in Tanjung Luar Fish Landing Port, East Lombok, Indonesia. AACL Bioflux.

[62] Tsikliras A.C., Dimarchopoulou D. (2021). Filling in Knowledge Gaps: Length–Weight Relations of 46 Uncommon Sharks and Rays (Elasmobranchii) in the Mediterranean Sea. Acta Ichthyol. Piscat..

[63] Maynou F., Sbrana M., Sartor P., Maravelias C., Kavadas S., Damalas D., Cartes J.E., Osio G. (2011). Estimating Trends of Population Decline in Long-Lived Marine Species in the Mediterranean Sea Based on Fishers’ Perceptions. PLoS ONE.

[64] Fortibuoni T., Borme D., Franceschini G., Giovanardi O., Raicevich S. (2016). Common, Rare or Extirpated? Shifting Baselines for Common Angelshark, *Squatina squatina* (Elasmobranchii: Squatinidae), in the Northern Adriatic Sea (Mediterranean Sea). Hydrobiologia.

[65] Cavanagh R.D., Gibson C. (2007). Overview of the Conservation Status of Cartilaginous Fishes (Chondrichthyans) in the Mediterranean Sea.

[66] IUCN (2010). IUCN Red List of Threatened Species.

[67] European Commission (2009). The Protection of Species of Wild, Amending Council Regulation (EC) No 338/97.

[68] European Commission (2003). The Removal of Fins of Sharks on Board Vessels.

[69] Dulvy N.K., Baum J.K., Clarke S., Compagno L.J.V., Cortés E., Domingo A., Fordham S., Fowler S., Francis M.P., Gibson C. (2008). You Can Swim but You Can’t Hide: The Global Status and Conservation of Oceanic Pelagic Sharks and Rays. Aquat. Conserv..

[70] Bradai M.N., Saidi B., Enajjar S. (2010). Elasmobranchs of the Mediterranean and Black Sea: Status, Ecology, and Biology Bibliographic Analysis. GFCM Meeting Document, Transversal Expert Meeting on the Status of Elasmobranchs in the Mediterranean and the Black Sea Tunisia, Salammbô.

[71] Farrag M.M.S., Mustafa A.A., Abdelazim I.M., Osman Y.A. (2025). Vulnerable Marine Vertebrates along the Egyptian Mediterranean Coast: Challenges to the Anthropogenic, Fisheries Bycatch and Climatic Changes. Endangered Marine Vertebrates—Recent Advances for Conservation.

[72] GAFRD (2012). General Authority for Fish Resources Development. Fish Statistics Yearbook.

